# Creation of a novel simulation based palliative care curriculum for the emergency medicine resident

**DOI:** 10.1186/s12909-026-09503-1

**Published:** 2026-05-25

**Authors:** Hillary C. Moss, Bruce R. Gutierrez, Mustfa K. Manzur, Amir L. Rashed, John J. Thomas, Noah P. Trump, Danielle K. Turrin, Valerie C. Lehman, Michael Cassara, Carlo L. Lutz

**Affiliations:** 1 Albert Einstein College of Medicine, Bronx, NY USA; 2 Montefiore Medical Center, Bronx, NY USA; 3 Weill Cornell Medical College, New York, NY USA; 4 New York-Presbyterian Weill Cornell Medical Center, New York, NY USA; 5 New York City Health + Hospitals Jacobi Medical Center, Bronx, NY USA; 6 University of Texas Southwestern Medical School, Dallas, TX USA; 7 University of Texas Southwestern Medical Center, Dallas, TX USA; 8 Jack D. Weiler Medical Center, Bronx, NY USA; 9 Donald and Barbara Zucker School of Medicine at Hofstra/Northwell, Hempstead, NY USA; 10 North Shore University Hospital, Manhasset, NY USA; 11 Garnet Health Medical Center, Middletown, NY USA

**Keywords:** Curriculum, Palliative care, Emergency medicine, Simulation, Medical education

## Abstract

**Background:**

Palliative Care focuses on improving quality of life and preventing suffering, especially near the end of life. With an aging population that frequently seeks care in the Emergency Department (ED), it is crucial for Emergency Medicine (EM) physicians to be well versed in the skills needed for palliative care. However, many EM residency programs lack formal training in this area. This study aimed to develop, implement, and assess a year-long, simulation-based palliative care curriculum.

**Methods:**

This IRB-exempt observational cohort study was conducted from July 2023 to June 2024 at a large urban EM residency program. A group of content experts in palliative care and simulation-based education designed the curriculum utilizing the Kern model of curriculum development, integrating simulation, small group discussion, and didactic lectures. The curriculum was delivered episodically throughout the academic year during regular residency didactics. The curriculum’s impact on knowledge, attitudes, and practice patterns regarding palliative care was assessed using pre- and post- intervention surveys.

**Results:**

Fourteen EM residents completed both pre- and post-intervention surveys. There was a statistically significant improvement in self-reported practice patterns (35.7% to 60.7% *p* = 0.0055), but no significant change in attitudes (73.4% to 73.4% *p* = 0.5020) or knowledge (48.9% to 57.1% *p* = 0.0758).

**Conclusions:**

A year-long simulation-based palliative care curriculum significantly improved EM residents’ practice patterns in regard to palliative care, with a positive trend in knowledge retention. Feasibility of implementation was demonstrated. Findings suggest that simulation-based training effectively teaches key palliative care skills. This curriculum may serve as a model for integrating palliative care education into other EM residency programs and may improve resident preparedness in caring for critically ill patients near the end of life.

**Supplementary Information:**

The online version contains supplementary material available at 10.1186/s12909-026-09503-1.

## Introduction

Palliative Care is an approach to medicine that focuses on quality of life and prevention of suffering of all kinds, whether physical, psychosocial, or spiritual [1]. It is recognized by the Institute of Medicine as an integral component of the practice of any clinician who treats patients near the end of life [2]. Given that more than 75% of older adults visit the emergency department (ED) in the last six months of life, it is imperative that emergency medicine (EM) clinicians have a strong understanding of palliative care [3]. The combination of the COVID-19 pandemic, prolonged ED boarding times, and the overall aging of the American population has led to an influx of medically complex patients with diverse healthcare needs [4, 5]. Many of these patients present without formal advanced directives, which means that, in critical situations, the default treatment approach involves aggressive and invasive measures, such as intubation and CPR [6, 7]. These interventions may not align with the patient’s goals of care, often leading to unnecessary suffering [8]. Having the skills, confidence, and knowledge to tailor interventions to a patient’s actual priorities can help emergency physicians provide more thoughtful and compassionate care, ultimately changing the trajectory of a patient’s entire hospital course. Although the importance of palliative care in EM is clear, the effectiveness of palliative care training for emergency physicians remains uncertain [9]. One study found that while most EM residents recognized the importance of developing palliative care skills, many felt unprepared to implement these skills and engage in goals of care discussions [10]. Additionally, only 38.5% of EM program directors report having structured end-of-life curricula, with significant variability and a lack of standardization [11]. While there have been efforts to create formal curricula for emergency residents in palliative care, these have often been limited to single-day sessions on one aspect of care (i.e., death notification simulations) or primarily asynchronous lecture-based models such as the Education in Palliative and End-of-life Care in Emergency Medicine (EPEC-EM) [3, 10–12]. Simulation has been successfully utilized in EM resident training and is widely accepted among residency educators due to its ability to provide hands-on practice in a controlled, low-risk environment.

Simulation-based teaching facilitates active learning, which has been associated with improved educational outcomes compared to traditional lecture-based instruction in both general education and simulation-based medical education specifically [13, 14]. While much of simulation-based education has focused on procedural skills and medical management, it has also been successfully utilized in teaching communication-based skills [15, 16]. The simulation-based palliative care education that exists focuses on standardized patient portrayals and communication skills in single-session interventions, often avoiding the clinical symptom management aspect of the field, and missing an opportunity to create a more holistic educational experience [17]. To date, no simulation-based longitudinal curricula have been created to teach EM residents the core tenets of palliative care including both the communication-based skills and the more clinical based symptom management skills.

We aimed to develop and implement a year-long palliative care curriculum integrating simulation, small group discussion, and didactic lectures, and to evaluate its effect on residents’ self-reported palliative care knowledge, attitudes, and practice-relevant behaviors. While prior educational models — including asynchronous curricula such as EPEC-EM and brief bootcamp formats — have shown incremental gains in knowledge and learner self-assessment, to our knowledge no published longitudinal simulation-based curriculum has addressed both communication skills and clinical symptom management for EM residents. We hypothesized that following our curriculum, residents would demonstrate measurable improvements across these three self-reported domains. We acknowledge that improvements in survey-based outcomes may not directly translate to changes in clinical behavior, and that objective outcome measures will be necessary in future work to establish clinical impact.

## Methods

We conducted an observational cohort pilot study of the impact of a simulation-based palliative care curriculum on the palliative care-centered knowledge, attitudes, and practice patterns of EM residents. The study was conducted at the Jacobi-Montefiore EM residency at Einstein College of Medicine, a large New York City based residency, from July 1, 2023, to June 30, 2024. This residency has access to a robust simulation center and has a strong simulation presence in residency didactics. All residents participate in multiple simulation experiences every month, including small group experiences with their PGY level, large group experiences with a mixed PGY cohort, and in situ simulations with a multidisciplinary team, depending on their clinical schedules and conference availability. All residents in the program were eligible to participate in the study; participation was voluntary. The IRB categorized the study as exempt human subjects’ research. This study was funded by the Society for Academic Emergency Medicine, Simulation Academy Novice Researcher Grant, AG2020-0000000134.

### Curriculum development

We used the Kern six-step model for curriculum development to design our educational intervention. We convened a multidisciplinary team of simulation and palliative-care content experts, to identify content aligned with learner-specific educational needs. Our team reviewed modules from the Center to Advance Palliative Care fellowship curriculum and the Education in Palliative and End-of-life Care in Emergency Medicine (EPEC-EM) [12] intensive curriculum and selected those that were felt to be most relevant to the EM Physician. We sought to identify those content domains most relevant to palliative care provided within the context of acute care in the ED. Seven core topics were chosen, and specific learning objectives were modified from the EPEC-EM curriculum to better align with the intended learning modalities and to guide the development of educational content. We then created simulation cases to highlight these core topics. The seven content domains and learning objectives are displayed in Table 1. Full case materials are provided in Supplementary File 1.

**Table 1:** Curriculum topics and learning objectives

Topic	Learning Objectives
Communicating Serious News	o Assessing patient and/or caregiver understanding of the diagnosis and prognosis o Getting permission to share serious news, o Communicating serious news clearly and compassionately o Confirming patient and family understanding and responding to emotional reactions
Goals of Care Discussions	• Eliciting patient/surrogates understanding of the patient’s condition and values • Guiding surrogates in exploring the patient’s values and goals • Defining the goal of advance care planning (ACP) conversations and distinguishing them from current decision making • Recognizing that ACP informs understanding of the patient’s values and enables non-threatening future care discussion.
End of Life Symptom Management	• Managing dyspnea with non-pharmacologic (e.g., pleural catheters, thoracentesis) and pharmacologic (e.g., oxygen, opiates, nebulized furosemide, nebulized opiates) strategies. • Managing constipation, especially as it relates to opiates, • Managing nausea and vomiting
Cancer Complications	• Recognizing and treating hypercalcemia, cord compression, pathologic fractures, and pleural effusions.
Pain Management	• Describing the appropriate use of various pain medications, including the minimum effective dose. • Identifying the appropriateness of opioid therapy and understanding the pharmacokinetics and dosing in opioid naive and dependent patients. • Understanding the use of reversal agents and medication for addiction treatment.
Legal & Ethical Issues	• Determining medical decision-making capacity • Differentiating healthcare proxies, surrogates, and power of attorney. • Describing the components of the MOLST form and its legal implications.
Hospice Care	• Differentiating between hospice and palliative care • Communicating with hospice staff when a patient presents in the ED • Managing uncontrolled symptoms from hospice • Identifying the process to refer to hospice from the ED.

We sequenced simulation cases to build upon one another, reinforce key communication skills, and ensure skill retention. For example, the first case focused on communicating serious news, but later cases incorporated this skill alongside other topics.

Content experts prepared lectures on each core topic, which were delivered on the same day as the related simulation case to reinforce the learning objectives. We implemented the curriculum longitudinally, with sessions spaced intermittently over the academic year to avoid excessive cognitive and emotional load (i.e., burn out) from frequent engagement with emotionally charged scenarios.

### Simulation based education & debrief

Simulation cases were run at the Institute of Medical Simulation and Advanced Learning (IMSAL) located on the Jacobi Hospital Campus as small group sessions embedded in residents’ weekly educational conferences. Residents were grouped into mixed developmental level groups of 4–5 residents each, with 2–3 residents participating as patient care providers in each case, while the remaining residents actively observed.

Each case offered residents three types of interactions.

1. Primary interactions with the patient, portrayed as a SimMan 3G mannequin operated by simulation operations specialists using LLEAP software (Laerdal Medical Corporation, Wappingers Falls, NY).
2. Secondary interactions with embedded participants (nurses, technicians, consultants) portrayed by IMSAL staff and simulation faculty.
3. Tertiary interactions with the patient’s family, portrayed by standardized participants trained in advance by the study team.

To improve realism and to address challenges presented by visitor restrictions due to the COVID-19 pandemic, some cases involved standardized family members participating via phone or video calls. Each case lasted a maximum of 15 min, followed by a 45-minute post-case debrief. Sessions occurred approximately every six weeks, yielding seven sessions over the academic year.

Post-case debriefs included all residents in attendance, two EM/Simulation Fellowship-trained attendings, one dual-trained EM-Palliative Medicine Attending or Fellow, and any Standardized patients who were involved in the given case. The Palliative Care team member served as a content expert throughout the discussion, with the Simulation faculty serving as the leaders of the discussion. Debriefs followed the the PEARLS (Promoting Excellence and Reflective Learning in Simulation) model, following a structured format of : a reaction phase for residents to process their emotions, followed by a deep dive into the scenario to identify areas where learners excelled (pluses) or were challenged and would alter how they performed if given the chance (deltas), concluding with learners sharing key takeaways from the case [18]. Standardized patients were recruited from the Albert Einstein College of Medicine SP program, where they previously have undergone PEARLS-based debriefing training, and they participated as co-debriefers in these discussions, focusing feedback to residents on their communication skills. Debriefs focused both on medical management and communication based skills, with the later incorporating established frameworks (e.g. SPIKES for delivering serious news). The details of these frameworks are provided within the case debrief scipts in Supplementary File 1. The PEARLS framework and debriefing tool are additionally provided in Supplementary File 2.

### Survey creation

We used a pre-post intervention survey design to evaluate the effectiveness of the educational intervention on residents’ knowledge, attitudes, and practice patterns regarding palliative care in EM. Outcome domains were measured with an adapted version of the validated Palliative Care Attitude and Practice Survey [19] (Al-Ansari et al.,). Adaptations served to better reflect the audience taking the survey as the original survey was multidisciplinary and built towards the Kuwaiti structure of medical education. Additionally knowledge retention questions were added to ensure all sessions had content reflected on the survey. The survey was adapted from a previously published tool assessing palliative care attitudes and knowledge among physicians in Kuwait and was reviewed for clarity and relevance by the content expert team. The adapted instrument included sixteen knowledge items, seven attitude items, and four practice pattern items. The instrument as reviewed for content validity by the multidisciplinary content expert team prior to administration. The full survey instrument is provided in Supplementary File 3.

The survey included three sections that align with the three goals of the curricular intervention:

1. Knowledge
  Multiple choice questions testing core palliative care topics relevant to EM. Responses were scored as correct or incorrect, and a cumulative score was calculated to assess overall knowledge.
2. Attitudes
  Questions aimed to assess residents’ perspectives on the importance and feasibility of providing palliative care in the ED. Responses were rated on a 5-point Likert scale(1 = strongly disagree to 5 = strongly agree).
3. Practice Patterns
  Questions assessed residents’ self-assessed practice patterns in utilizing palliative care in the ED, with responses rated on a 5-point Likert scale (1 = never to 5 = always).

A convenience sample of residents across all post-graduate year (PGY) levels completed the survey at two time points: immediately prior to the intervention (pre-intervention survey) and 3 months post intervention (post-intervention survey). Standard informed consent procedures were followed, and study participation was completely voluntary and anonymous, with de-identified responses.

### Statistical analysis

Statistical analysis was conducted using STATA v16. The primary outcome was survey scores stratified by the domains above, reported as a percentage of correct or positive answers, which were measured pre-and post-intervention. Survey responses were scored on a scale of 0–100%. Likert scale responses were dichotomized into high scores (4–5) and low scores (1–3); scores of 3 were categorized as low as they do not reflcet active adoption of palliative are principles. The primary outcome was assessed using paired t test, or a non-parametric equivalent if data collected were not normally distributed.

The difference between pre-and post-intervention scores was also calculated. To determine if there was a correlation between participant characteristics and change in test scores, bivariate associations between the pre/post intervention change in score and participant demographic information was calculated. Age, sex, and PGY year were selected apriori for biological plausibility.

Multiple regression analysis to investigate the bivariate associations above was initially planned, but deferred as the final sample size did not meet the sample size requirement of 10 participants per independent variable, particularly in light of the likely collinearity between age and PGY year.

## Results

### Participant characteristics

Out of 84 available EM residents, a total of 14 EM residents completed the study. As shown in Table 2, the demographics were as follows: 57% female (*n* = 8), 43% male (*n* = 6), 85% MD (*n* = 12), 15% DO (*n* = 2), 7% PGY1 (*n* = 1), 42% PGY2 (*n* = 6), 21% PGY3 (*n* = 3), 28% PGY4 (*n* = 4). The mean age of participants was 32 years old (SD 5.1).

**Table 2:** Participant (resident) characteristics (*n* = 14)

Age (y/o)*		29 (28–35)
Gender **	Female	8 (57%)
	Male	6 (43%)
Degree**	MD	12 (85%)
	DO	2 (15%)
PGY level **	1	1(7%)
	2	6 (42%)
	3	3 (21%)
	4	4 (28%)

*Represented as median (QR)

** Represented as n (%)

### Pre and post intervention comparison

The pre- and post- intervention survey results, broken down into the three domains of practice patterns, attitudes, and knowledge of palliative care, are summarized in Table 3. Non-parametric Wilcoxon tests were used due to non-normal distribution of the data. The average score in the practice patterns domain increased from 35.7% pre-intervention to 60.7% post-intervention (*p* = 0.0055). The average scores in the attitudes domain were 73.4% pre-intervention and 73.4% post intervention; despite similar group-level means, therew as a statistically significant difference on paired analysis (*p* = 0.02), reflecting variability in individual trajectories across the intervention The average score in the knowledge domain increased from 48.9% pre-intervention to 57.1% post-intervention (*p* = 0.0758).

**Table 3:** Pre and post intervention comparisons (*n* = 14)

Domain	Pre-Intervention Average Score(%)	Post-Intervention Average Score (%)	P-Value
Practice Patterns	35.7%	60.7%	**0.0055**
Attitudes	73.4%	73.4%	**0.020**
Knowledge	48.9%	57.1%	0.0758

### Comparison of domain score changes with demographics

Data analysis was performed to analyze the association between changes in scores across the three survey domains with participant demographics (PGY level, age, and gender) and is summarized in Table 4. Spearman’s correlation was used to analyze the relationship between the change in survey scores (Practice Patterns, Attitudes, and Knowledge) and PGY level (*p* = 0.38; 0.48; 0.87) and age (*p* = 0.58; 0.98; 0.98), while Wilcoxon tests were used to analyze the relationship between the change in survey scores and gender (*p* = 0.63; 0.01; 0.05).

**Table 4:** Change in domain score stratified by participant demographics

	PGY Year					Age		Gender		
	1	2	3	4	*p*	rho	*p*	M	F	*p*
Practice Patterns	50%	25%	33%	13%	0.3767	16%	0.5803	21%	28%	0.6324
Attitudes	14%	10%	10%	4%	0.4848	1%	0.9763	10%	7%	0.0091
Knowledge	38%	3%	5%	12%	0.8653	1%	0.9753	17%	2%	0.0476

Subgroup sample sizes: Male *n*=6, Female *n*=8; PGY1 *n*=1, PGY2 *n*=6, PGY 3 *n*=3, PGY4 *n*=4

## Discussion

Palliative Care is an integral aspect of EM, especially as our population ages, becomes more chronically ill, and faces longer ED boarding times. As the American Board of Emergency Medicine (ABEM) transitions its oral certification exam to focus more on the key communication skills needed for palliative care, the importance of improving emergency physician education in this critical domain becomes more apparent. This intervention aimed to address the gap in palliative care education by providing hands-on training through standardized patient-augmented simulation-based education.

### Effectiveness of the curriculum

These pilot results suggest that the curriculum was effective. There was a statistically significant improvement in resident self-reported practice patterns following the intervention, suggesting residents perceived greater readiness to engage in palliative care behaviors following the curriculum. Whether this translates to observable clinical practice change warrants evaluation with objective measures such as chart-triggered audits. These reported improvements also suggest that simulation-based education was a successful tool for teaching essential communication skills in palliative care. The active learning environment- where residents practiced skills and received immediate feedback from both trained debriefers and standardized patients- may have facilitated residents’ readiness to engage in goals of care conversations, deliver death notifications, and other challenging communications. This hands-on approach may have facilitated the integration of these skills into their clinical practice.

There was a positive trend towards improved knowledge of key palliative care topics, although this did not reach statistical significance. This could be attributed to the underpowered nature of the study or to the fact that the knowledge questions did not fully capture the breadth of the content within the curriculum. It is also possible that residents missed questions related to topics and simulations in which they were not an active participant, and that active participation may be important in knowledge retention as well. Interestingly, despite a lack of change in overall attitudes toward palliative care post-intervention, there was a statistically significant difference pre and post intervention on paired analysis. This likely reflects variability in individual level trajectories across the intervention. Additionally, given the unique challenges of the ER practice environment- where residents often board critically ill patients in the ED for extended periods- attitudes toward palliative care may have already been highly favorable, leaving little overall room for improvement.

An interesting and unexpected finding was the statistically significant change in attitudes and knowledge when data were broken down by gender. Female residents exhibited statistically significant improvement in both attitude and knowledge post-intervention which raises questions about potential gender-specific differences in engagement with palliative care education. However, given the very small subgroup sizes (male *n* = 6, female *n* = 8), these findings should be interpreted with caution and may reflect sampling variability rather than a true effect. Future studies with larger samples are neeed to determine whether this represents a meaningful difference or an artifact of sample size.

### Comparison with prior literature

When placed in the context of prior studies, our curriculum aligns with and builds upon the existing literature. Previous studies focused on didactic asynchronous curricula have demonstrated some improvement in resident knowledge of palliative care, while previous studies focused on simulation-based medical education (typically limited to one aspect of palliative care)have demonstrated some improvement in resident practice patterns of palliative care [10, 12, 20]. A more recent study made progress towards a more intensive and broad curriculum, with a one month long primarily didactic approach with a capstone of a simulation session to practice skills [21]. This bootcamp was deemed successful, but focused solely on residents self reporting improvement in their skills, and ultimately this curriculum was limited by being presented to a single PGY class, over one distinct point in time. Our study builds on the literature, combining didactic and simulation-based approaches, longitudinally over multiple time points and multiple PGY classes to create a comprehensive curriculum that addresses not only communication skills but also clinical aspects of palliative care. The survey data aligns with the previous literature as well, demonstrating improved resident knowledge and practice patterns as related to palliative care. The combination of didactics for key knowledge acquisition, active learning for skills acquisition and feedback, and spaced repetition for skill revisiting and solidification is the key to the success of this curriculum. The ability to teach all of these components within a single curriculum makes it easier for residency programs to incorporate palliative care into an already crodwed education schedule.

### Limitations

This study is limited in large part by the small sample size of residents who were able to complete both pre- and post- intervention surveys. Only residents who completed both the pre- and post-session surveys (*n* = 14) were including in paired analyses. Although 48 residents completed either the pre- or post-survey, pairing limited statistical power. The blinding process prevented us from identifying missed pre-surveys, and precluded ad-hoc post-surveys. Item-level survey response data were not retained in a format that permitted post-hoc calculation of internal consistency; future implementations of this curriculum should prospectively report Cronbach’s alpha for each domain to strengthen themeasurement validity evidence base. Additionally, given the small paired smaple size (*n* = 14), effect size estimates and confidence intervals for the primary outcomes would carry substantial uncertainty; future studies with larger samples should report these alongside p-values to facilitate intepretaiton.

Recruitment was challenging due to the unpredictable nature of resident schedules, making it difficult to ensure that the same residents were available on the days that the experimental curriculum was being delivered. As a result, the study’s ability to assess the full effectiveness of the curriculum is limited. However, the data collected does suggest that the curriculum was beneficial and demonstrates its feasibility. Furthermore, the success of the initial implementation has sparked interest from other residency programs, and discussions are ongoing to expand the curriculum to additional sites.

An additional limitation of this study is that residents self-reported their practice patterns in their survey responses. Future studies should incorporate chart-triggered audits for objective corroboration.

The high attrition rate (14/84 residents completing paired surveys) warrants further explanation. Attendance was determined by each resident’s clinical schedule on the days sessions were embedded in protected conference time — a well-recognized structural constraint in residency education research. Notably, 48 residents completed at least one survey, suggesting broad curricular engagement; the paired sample limitation reflects scheduling logistics rather than disengagement. These findings have direct implications for feasibility and sustainability. Programs considering adopting this curriculum should anticipate variable attendance driven by scheduling, and should consider building sessions into a recurring multi-year cycle to maximize cumulative resident exposure. Critically, the favorable results observed in this small paired sample support the value of continued iteration rather than suggesting the curriculum is ready for wide dissemination without further study.

It is important to recognize that this pilot study occurred in a high resource residency program with access to high fidelity simulation and standardized patients. Programs lacking a simulation center can substitute low-fidelity role-play or virtual standardized patients; required material and scripts are included in Supplementary File 1.

Future work will focus on refining and expanding the curriculum, with plans to create a freely accessible repository of simulation cases and curriculum materials for use by residency programs nationwide. This work will also serve to recruit a broader variety of residencies (urban vs. rural, geographic diversity) to allow for broader generalizability of the findings. By increasing the sample size and improving the diversity of participating institutions, future research will provide a stronger basis for more meaningful statistical analysis. Additional future work should directly compare longitudinal and single-session models to determine the optimal balance of efficacy and resource utilization.

Finally, despite these limitations, the study reinforces the importance of palliative care education in EM and underscore the potential for simulation-based curricula to effectively integrate key communication skills into residency training.

## Conclusions

This study successfully developed and implemented a year-long simulation-based palliative care curriculum at a single urban EM residency. The curriculum led to significant improvements in residents’ self-reported palliative care behaviors and demonstrated a positive trend toward increased knowledge of key palliative care topics. These findings suggest that simulation-based education is a promising method for teaching EM residents the essential skills required for effective palliative care.

Given these results, we recommend that residency programs consider incorporating active learning interventions, such as simulation-based palliative care curricula, into their educational framework. Such initiatives have the potential to improve residents’ communication skills and overall preparedness for managing patients with serious, life-limiting illnesses in the ED.

## Supplementary Information

Supplementary Material 1.

Supplementary Material 2.

Supplementary Material 3.

## Data Availability

Copies of the simulation cases utilized in this work are included as an appendix. Underlying data gathered in this study can be shared by request to the corresponding author.
