# Supplementary material for: Creation of a novel simulation based palliative care curriculum for the emergency medicine resident
Source: BMC Med Educ. 2026 May 25;26:1175. doi: 10.1186/s12909-026-09503-1 (PMC13383450; doi:10.1186/s12909-026-09503-1)
Supplement: Supplementary file 1 — Supplementary Material 1. [file 12909_2026_9503_MOESM1_ESM.docx]

**Section 1: Case Summary**

| **Scenario Title:** | **Hypercalcemia of Malignancy/ GOC Conversation** |
| --- | --- |
| Keywords: | Hypercalcemia, malignancy, palliative care, oncologic emergencies |
|  |  |
| Brief Description of Case: | 67yo M with PMH presents for confusion, fatigue, and nausea and is found to have hypercalcemia from new diagnosis of advanced malignancy. Symptoms improve after being given intravenous hydration. |

| **Goals and Objectives** | |
| --- | --- |
| Educational Goal: | Recognize and manage hypercalcemia in the cancer patient |
| Objectives: | 1. Create a wide differential diagnosis for altered mental status 2. Recognize signs and symptoms of hypercalcemia 3. Initiate treatment for hypercalcemia of malignancy 4. Deliver bad news in a patient with advanced malignancy 5. Perform an empathic GOC conversation with a surrogate decision maker |

| **Learners, Setting and Personnel** | | | | | |
| --- | --- | --- | --- | --- | --- |
| Target Learners: | ☒ Junior Learners | | ☒ Senior Learners | | ☐ Staff |
|  | ☐ Physicians | ☐ Nurses | | ☐ RTs | ☐ Inter-professional |
|  | ☐ Other Learners: | | | | |
| Location: | ☒ Sim Lab | | ☐ In Situ | | ☐ Other: |
| Recommended Number of Facilitators: | Instructors: 2 | | | | |
|  | Sim Actors:2 | | | | |
|  | Sim Techs: 1 | | | | |

**Section 2A: Initial Patient Information**

| 1. **Patient Chart** | | | | | | |
| --- | --- | --- | --- | --- | --- | --- |
| Patient Name:  Miguel Rodriguez | | | | Age: 67 | Gender: M | Weight: 75 kg |
| Presenting complaint: Confusion, nausea, fatigue | | | | | | |
| Temp: 38.5 | HR:  130 | BP:  88/62 | | RR: 28 | O_2_Sat: 93% | FiO_2_:RA |
| Cap glucose: 150 | | | | GCS: (E V M ) 13 | | |
| Triage note:  67 yo M w/ HTN, DM, HLD, COPDr presents to ED with wife for 4 days of worsening fatigue, confusion, nausea, and poor PO intake. | | | | | | |
| Allergies: NKDA | | | | | | |
| Past Medical History:   - HTN - DM - HLD | | | Current Medications:   - Losartan 100 mg QD - Hydrochlorothiazide 25 mg QD - Lipitor 40 mg QD - Metformin 1000 mg BID | | | |

**Section 2B: Extra Patient Information**

| **A. Further History** | |
| --- | --- |
| *Include any relevant history not included in triage note above. What information will only be given to learners if they ask? Who will provide this information (mannequin’s voice, sim actors, SP, etc.)?*  Patient’s wife will be at bedside and provide additional history. Per patient’s wife, patient gradually stopped eating and has been drinking less fluids over the past 4-5 days. He also seems “out of it” and would appear to not know the date or appears a lot more forgetful than usual. At baseline, patient is typically A+Ox3 and performs all IADLs independently. Review of systems remarkable for poor appetite, fatigue, nausea, constipation, and confusion. ROS otherwise negative. Wife notes that husband had been feelingunwell for a few weeks but really declined in the last few days . pt is stubborn and refused to go to doctor but now things are so bad that wife was able to get him to the doctor.. | |
| **B. Physical Exam** | |
| *List any pertinent positive and negative findings* | |
| Cardio: tachycardic, regular rhythm, no M/R/G | Neuro: A+Ox2 (oriented to person and place only), grossly non-focal |
| Resp:  mild diffuse wheezing, decreased bs at right base | Head & Neck: supple, no tenderness |
| Abdo: soft, nontender, no rebound, guarding, rigidity | MSK/skin: no rash, purpura, or ecchymosis |
| Other: dry mucus membranes | |

**Section 3: Technical Requirements/Room Vision**

| **A. Patient** |
| --- |
| ☒ Mannequin *(specify type and whether infant/child/adult): adult* |
| ☐ Standardized Patient |
| ☐ Task Trainer |
| ☐ Hybrid |
| **B. Special Equipment Required** |
| IV fluids |
| **C. Required Medications** |
| Normal saline, bisphosphonates |
| **D. Moulage** |
| None |
| **E. Monitors at Case Onset** |
| ☐ Patient on monitor with vitals displayed  ☒ Patient not yet on monitor |
| **F. Patient Reactions and Exam** |
| *Include any relevant physical exam findings that require mannequin programming or cues from patient*  *(e.g. – abnormal breath sounds, moaning when RUQ palpated, etc.) May be helpful to frame in ABCDE format.*  A – protecting airway, moaning, confused  B – wheezing in all lung fields, decreased BS at right base  C – tachycardic, no hemorrhage  D – follows some commands, opens eyes to verbal stimuli  E – no signs of trauma, no wounds |

**Section 4: Sim Actor and Standardized Patients**

| **Sim Actor and Standardized Patient Roles and Scripts** | |
| --- | --- |
| *Role* | *Description of role, expected behavior, and key moments to intervene/prompt learners. Include any script required (including conveying patient information if patient is unable)* |
| Family member:  wife | “I’m not sure what’s going on with him. He was fine just the other week, but now he just seems confused. He also doesn’t want to eat his dinner and just plays with his food, which is weird because he loves my cooking.” |

**Section 5: Scenario Progression**

| **Scenario States, Modifiers and Triggers** | | | | |
| --- | --- | --- | --- | --- |
| Patient State/Vitals | Patient Status | Learner Actions, Modifiers & Triggers to Move to Next State | | Facilitator Notes |
| **1. Baseline State**  Rhythm: sinus tachycardia  HR: 130  BP: 88/60  RR: 28  O_2_SAT:93 %  T: 38.5^o^C  GCS: 12 | Confused, dehydrated, appears weak, ill appearing  moaning | Expected Learner Actions  ☐ Send labs  ☐ Obtain FSG  ☐ Obtain EKG  ☐ Send urine  ☐ Attempt to obtain head CT  ☐ Place patient on supplemental oxygen  ☐ | Modifiers  *Changes to patient condition based on learner action*  - tachycardia will gradually improve after IVF started  -  Triggers  *For progression to next state*  -  - | If residents ask for head CT, tell them they are unavailable due to a prolonged break.  CXR will show R sided pleural effusion, tumor, bony metastasis |
| **2.**  Rhythm: Sinus tachycardia  HR: 115  BP: 90/60  RR: 28  O_2_SAT:93%  T: 38.5^o^C  GCS: 12 | Stable no improvement of mental status | Expected Learner Actions  ☐ Reassess volume status  ☐ Reattempt to obtain head CT  ☐ Obtain CXR  ☐ Start bisphosphonates IV  ☐ Admit to telemetry  ☐ Discontinue HCTZ | Modifiers  - pt’s oxygen saturation will improve if placed on supplemental oxygen but his work of breathing will still be increased  -  -  Triggers  -  - | Only provide additional labs like PTH or PTHrP if specifically requested by residents  Can have patient go to CT scan quickly if residents insist. The CTH will show metastasis.  CXR will show R sided pleural effusion, tumor, bony metastasis |
| **3.**  Rhythm: normal sinus  HR: 101  BP: 100/65  RR: 28  O_2_SAT:95 %  T: 37^o^C  GCS: 14 | Stable | Expected Learner Actions  ☐ Explain lab results to wife  ☐ Explain that hypercalcemia indicates poor prognosis  ☐  ☐  ☐ | Modifiers  -  -  -  Triggers  - Wife will ask about lab results  - Wife will ask what this means regarding prognosis | Wife will prompt team to discuss GOC– want to know what the diagnosis is what the prognosis is can the pt  be cured  what are our options? |

**Appendix A: Laboratory Results**

| CBC   WBC 11.5   Hgb 12   Plt 150  Lytes   Na 146   K 3.4   Cl 110   HCO_3_ 21   AG 16   Urea 15   Cr 1.1   Glucose 150  Extended Lytes   Ca 15.5 mg/dL   Mg 1.5   PO_4_ 2.2   Albumin 3.5   TSH wnl   Intact PTH 8 pg/mL (ref: 10 – 65 pg/mL)   PTHrP 10 pmol/L (ref: < or = 4.2 pmol/L)  1,25 (OH)_2_D 60 ng/mL (ref: 50 – 70 ng/mL)  VBG   pH 7.41   pCO_2_ 38   pO_2_ 40   HCO_3_ 22   Lactate 1.9 | Cardiac/Coags   Trop wnl   D-dimer wnl   INR wnl   aPTT wnl  Biliary   AST wnl   ALT wnl   GGT wnl   ALP 150   Bili wnl   Lipase wnl  Tox   EtOH neg   ASA neg   Tylenol neg   Dig level neg   Osmols wnl |
| --- | --- |

**Appendix B: ECGs, X-rays, Ultrasounds and Pictures**

| 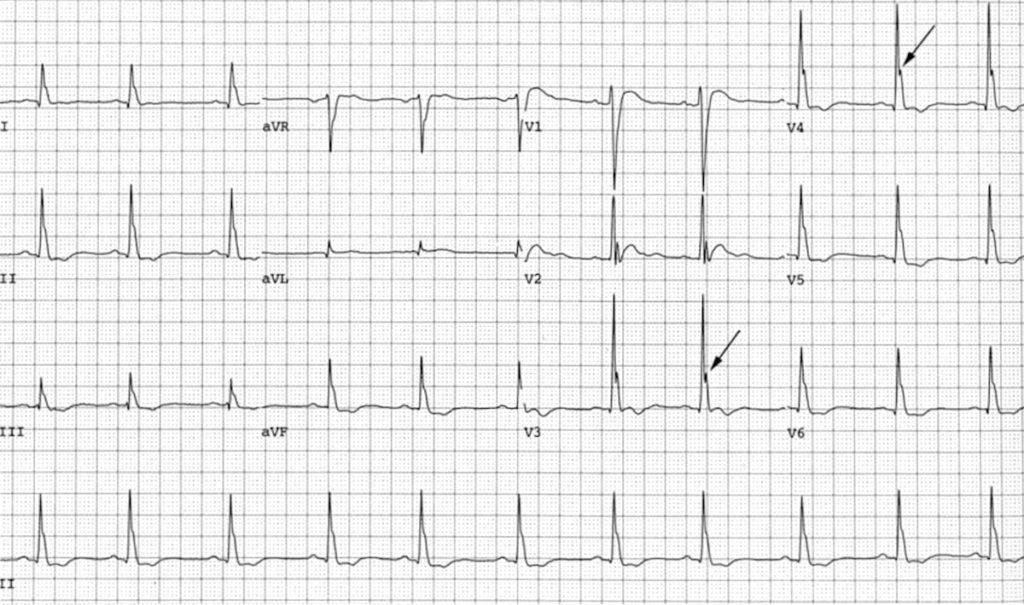  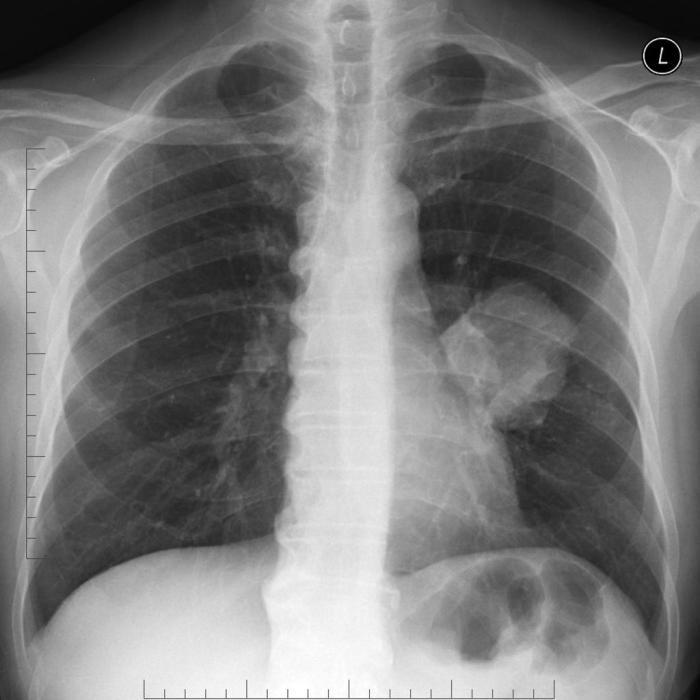  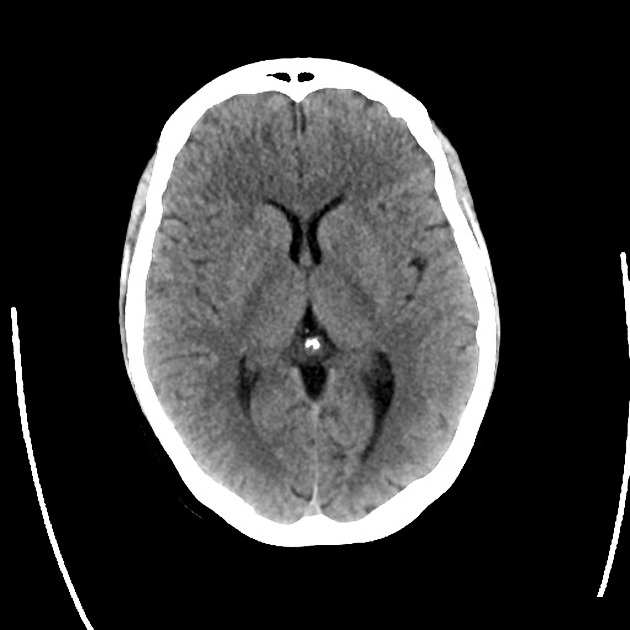 |
| --- |

**SP Case Notes**

**Goals of Care Conversations: Hypercalcemia of Malignancy**

| **Patient Name and DOB** | **Christopher Walker, 67 yo M**  **SP will play role of patient’s wife, Laura Walker** |
| --- | --- |
| **Opening Statement/**  **Chief Complaint** | **“My husband hasn’t been his normal self”** |
| **“Tell Me More About It”** | “He just seems out of it. He’s been slowly looking more confused over the last three months, it started with small things like forgetting to feed the dog. I noticed a few past due bills over the last month or so which is very unlike him, he’s normally on top of paying things on time.It got worse over the last week; he’s so tired, he won’t get out of bed. He’s also confused, he keeps calling out for our son but our son hasn’t lived with us in 20 years. He’d be so embarrassed if I told you this but he started wetting the bed this week as well, which he never does” |
| **Demeanor/ Physicality** | ***Concerned but calm, open body language towards providers*** |
| **History of Present Illness** | 67yo Male, current smoker, history of COPD, presents for confusion, fatigue, and nausea and is found to have hypercalcemia  You will be at the bedside and provide additional history. Your spouse gradually stopped eating and has been drinking less fluids over the past 4-5 days. He also seems “out of it” and does not know the date and appears a lot more forgetful than usual. At baseline, he is typically alert and oriented to person, place, and time and performs all activities of daily living independently. Review of systems remarkable for poor appetite, weight loss, fatigue, nausea, constipation, and confusion.. |
| **Past Medical Hx** | Hypertension, Diabetes Mellitus , Hyperlipidemia COPD |
| **Past Surgical Hx** | L knee arthroplasty, Hernia repair |
| **Allergies** | Penicillin |
| **Medications** | Ipratropium/Albuterol inhaler, Metformin, Losartan, Lipitor, HCTZ |
| **Over the Counter/ Vitamins & Supplements** |  |
| **Social Hx** | 30 Pack per day smoker, still smokes |
| **Family Medical Hx** | Breast Cancer in maternal aunt, father with heart disease |
| **ROS/Physical Findings** | **BP: 88/62 HR: 130 SpO2: 93% RR: 28 Temp: 38.5**  **General: Confused, older appearing man, ill appearing**  **Head eyes ear nose throat: dry mucous membranes**  **Cardio: Tachycardia**  **Pulmonary: Mild diffuse wheezing, Decreased lung sounds at Right lung base**  **Abdomen: Soft Nontender**  **Extremities: No edema** |
| **Props/ Moulage** |  |
| **SP Special instructions** | You have noticed your spouse’s decline for months, but they were stubborn and refused to come to the doctor. “he’s so stubborn! He didn’t want to leave home, I made the decision because he was so out of it”  Initial labs will show hypercalcemia, medical workup will reveal metastatic cancer (likely lung cancer secondary to his long smoking history). He will also have fluid buildup at his right lung (secondary to the cancer). After learning about the cancer/hypercalcemia:  “Is this because I’ve been feeding him too much milk?”  “Is he going to die? How long does he have to live?”  “What do we do next?”  The medical team should ask you what your spouse’s wishes would be in terms of advanced life support (intubation/breathing machine, chest compressions, life sustaining medications etc). Your spouse is too sick and out of it to be able to make these decisions.  *One of the main objectives of this case is for the team to have a goals of care conversation with you. If they do not prompt you to have this discussion you should bring it up “how likely is it that they make it out of this okay; what options do I have in terms of helping them” if they bring up intubation you can ask if its painful, if he will ever breathe on his own again”*  You should be extremely against any thoughts of him dying at first, the medical team should be able to give her reassurance that they are focused on his symptoms and his pain, the medical team should be gentle and caring towards you. Eventually, you will decide that you want everything but intubation because “he had a brother who was on life support after a bad car accident, he said he never wanted that to happen to him if it came down to it” |
| **Door Chart** |  |

**Section 1: Case Summary**

| **Scenario Title:** | **Metastatic Spinal Cord Compression** |
| --- | --- |
| Keywords: | Aortic dissection, chest pain, back pain, extremity weakness, oncologic emergencies, spinal cord compression, hypertensive emergency |
| Brief Description of Case: | 52 yo F w/ PMH poorly controlled HTN and recently diagnosed breast cancer here for chest pain, back pain, and left upper and lower extremity weakness and numbness. Patient presents markedly hypertensive, uncomfortable, and appears to initially have an aortic dissection. However, further imaging will reveal she has metastatic disease to the spine, causing her neurologic deficit. Patient will not have an aortic dissection but will show evidence of metastatic disease to the lower cervical/upper thoracic spine.  Radiation oncology will be consulted for emergent radiation therapy. |

| **Goals and Objectives** | |
| --- | --- |
| Educational Goal: | Identify and treat metastatic spinal cord compression in a cancer patient |
| Objectives: | 1. Recognize life-threatening causes of chest and back pain 2. Treat hypertensive emergency 3. Order appropriate labs and imaging in a patient with concerning chest and back pain 4. Advocate for patients and escalate issues to hospital administration quickly* 5. Recognize metastatic spinal cord compression as a potential cause of back pain in the cancer patient 6. Consult radiation oncology immediately upon diagnosis of metastatic spinal cord compression   *optional |

| **Learners, Setting and Personnel** | | | | | |
| --- | --- | --- | --- | --- | --- |
| Target Learners: | ☒ Junior Learners | | ☒ Senior Learners | | ☐ Staff |
|  | ☐ Physicians | ☐ Nurses | | ☐ RTs | ☐ Inter-professional |
|  | ☐ Other Learners: | | | | |
| Location: | ☒ Sim Lab | | ☐ In Situ | | ☐ Other: |
| Recommended Number of Facilitators: | Instructors: 2 | | | | |
|  | Sim Actors:2 | | | | |
|  | Sim Techs: 1 | | | | |

**Section 2A: Initial Patient Information**

| 1. **Patient Chart** | | | | | | |
| --- | --- | --- | --- | --- | --- | --- |
| Patient Name:  Melissa Grodskaya | | | | Age: 52 | Gender: Female | Weight: 65kg |
| Presenting complaint: chest and back pain | | | | | | |
| Temp: 98.7 | HR: 110 | BP: 220/150 | | RR: 26 | O_2_Sat: 98% | FiO_2_:RA |
| Cap glucose: 100 | | | | GCS: (E V M ) 15 | | |
| Triage note:  52 yo F w/ recently diagnosed breast cancer and HTN here for severe chest and back pain x 2 days. Patient also complaining of worsening left hand weakness, left arm numbness, and feeling more clumsy than usual including several trips without falling. Reports compliance with blood pressure medications. | | | | | | |
| Allergies: NKDA | | | | | | |
| Past Medical History:   - HTN - Breast Cancer (diagnosed 3 weeks ago; one round of chemotherapy) | | | Current Medications:   - Lisinopril 40 mg daily - Amlodipine 10 mg daily - Naproxen 250 mg BID | | | |

**Section 2B: Extra Patient Information**

| **A. Further History** | |
| --- | --- |
| *Include any relevant history not included in triage note above. What information will only be given to learners if they ask? Who will provide this information (mannequin’s voice, sim actors, SP, etc.)?*  Patient reports severe, worsening left-sided chest pain, non-exertional, non-pleuritic that radiates to her upper back worse with twisting movements and raising her arm. Has tried taking Tylenol and motrin without any relief. Also noted that over the past several days her left hand gradually felt weak and has trouble grasping objects. Also reports numbness to her left upper extremity that’s been worsening. Increasing clumsiness, dropping objects, feels like she is always tripping lately. No trauma or falls. No urinary retention, incontinence, saddle anesthesia, hx of IVDU. ROS positive for occasional SOB, anxiety, lightheadedness, nausea, but no vomiting. ROS otherwise negative. Recently diagnosed with breast cancer and underwent first round of chemotherapy about 2 weeks ago with pending mastectomy and radiation. No history of toxic habits. | |
| **B. Physical Exam** | |
| *List any pertinent positive and negative findings* | |
| Cardio: Tachycardic, regular rhythm, no murmurs, rubs, or gallops | Neuro: CNII-XII intact, PERRL, EOMI  3/5 grip strength left hand, 4/5 LUE flexion/extension   5/5 right hand, 5/5 RUE, 4/5 LLE dorsiflexion  Slow but steady gait, no dysmetria w/ FTN. Normal rapid alternating movements. |
| Resp: clear bilateral breath sounds | Head & Neck: supple, no tenderness |
| Abdo: soft, nontender. Non-distended, no rebound, guarding, rigidity. No CVAT. No pulsatile mass. | MSK/skin: no rash, purpura, or ecchymosis. +Mild left upper thoracic tenderness to palpation. No midline cervical, thoracic, or lumbar spine tenderness to palpation. No step offs. |
| General: Uncomfortable, grimacing, writhing around in pain, mildly diaphoretic | |

**Section 3: Technical Requirements/Room Vision**

| **A. Patient** |
| --- |
| ☒ Mannequin *(specify type and whether infant/child/adult): adult* |
| ☐ Standardized Patient |
| ☐ Task Trainer |
| ☐ Hybrid |
| **B. Special Equipment Required** |
| IV pump, manual BP cuff |
| **C. Required Medications** |
| Esmolol or other IV antihypertensive, morphine or other opioid, Zofran or other antiemetic |
| **D. Moulage** |
| None |
| **E. Monitors at Case Onset** |
| ☐ Patient on monitor with vitals displayed  ☒ Patient not yet on monitor |
| **F. Patient Reactions and Exam** |
| *Include any relevant physical exam findings that require mannequin programming or cues from patient*  *(e.g. – abnormal breath sounds, moaning when RUQ palpated, etc.) May be helpful to frame in ABCDE format.*  A – airway intact, speaking full sentences  B – clear bilateral breath sounds, no increased work of breathing  C – symmetric distal pulses, extremities well perfused  D – PERRL, A+OX3, decreased sensation to light touch LUE, 3/5 grip strength left hand, 4/5 LUE flexion/extension, 4/5 LLE dorsiflexion  E – no trauma, ecchymosis |

**Section 4: Sim Actor and Standardized Patients**

| **Sim Actor and Standardized Patient Roles and Scripts** | |
| --- | --- |
| Concerned Family Member | This person is a close family member of the patient.  After learning about concern for aortic dissection: “I just started supporting her/him through this breast cancer journey, we just started chemo, surgery is up next, but this back pain and high blood pressure was so sudden. I always get her/him to take their medication and on time, every time. How can this? We’re doing all the things the doctors said to do.”  After learning about metastases to the spine: “I just don't understand, breast cancer and now this? Our family doesn’t deserve this.”  The patient has always been so independent in their life, living on their own, traveling the world and things of that nature, will that continue to be the case? “Will they be able to live independently still?”  “How much time do we have left?” |

**Section 5: Scenario Progression**

| **Scenario States, Modifiers and Triggers** | | | | |
| --- | --- | --- | --- | --- |
| Patient State/Vitals | Patient Status | Learner Actions, Modifiers & Triggers to Move to Next State | | Facilitator Notes |
| **1. Baseline State**  Rhythm: sinus tachycardia  HR: 110  BP: 220/150  RR: 26  O_2_SAT:98 %  T: 37^o^C  GCS: 15 | Uncomfortable, crying, writhing around in pain | Expected Learner Actions  ☐ Start IV esmolol or other beta blocker  ☐ Obtain EKG  ☐ Call for STAT CTA of chest  ☐ Pain medication  ☐ Talk to radiology tech regarding emergent need for scan  ☐ Escalate to attending or administrator if rad tech pushes back  ☐ | Modifiers  *Changes to patient condition based on learner action*  - if no IV beta blocker started, patient will continue to have a rising BP  - if pain medication not given, BP will continue to be relatively high despite IV antihypertensive  Triggers  *For progression to next state*  - Blood pressure and heart rate will decrease after starting an appropriate dose of IV beta blocker  - CT scan will be done once the senior attending or department chair is contacted | Radiology tech can give push back about not having creatinine back and will request a note be written in the chart first.  After note is written, radiology tech will then continue to push back and say there are too many “STAT” patients that are ahead of this one and refuses to accept patient*  *optional |
| **2.**  Rhythm: normal sinus  HR: 70  BP: 150/90  RR: 20  O_2_SAT: 98%  T: 37^o^C  GCS: 15 | Will complain of nausea and started to vomit | Expected Learner Actions  ☐ Give antiemetic  ☐  ☐  ☐  ☐ | Modifiers  - if antiemetics aren’t given, patient will start to aspirate on vomit  -  -  Triggers  - ask for preliminary read CTA from radiologist  - | Preliminary read from radiologist will say no findings aortic dissection, but there will be evidence of bony metastasis in the cervical/thoracic vertebrae with possible surrounding edema. Only give this prelim read if requested. Learners may opt to just look at the CT scan themselves. |
| **3.**  Rhythm:  normal sinus  HR: 65  BP: 145/85  RR: 20  O_2_SAT:98 %  T: 37^o^C  GCS: 15 | Patient still complaining of severe pain. Will be distressed by the persistent numbness and weakness in arm and demand an explanation | Expected Learner Actions  ☐ Give additional pain medications  ☐Consult neurosurgery, radiation oncology  ☐give IV dexamethasone  ☐arrange for MRI spine  ☐feed patient if they are hungry | Modifiers  - if pain medication isn’t given, BP rises again despite antihypertensive therapy on board  - time permitting, patient will start to get impatient, tell the care team they are hungry and vociferously demand food  -  Triggers  - if food is not given, patient threatens to leave during evaluation  - | Patient will have mild improvement in pain, but will still be uncomfortable and have persistent left hand weakness. MRI results will not be available. Patient can eat. |

**Appendix A: Laboratory Results**

| CBC   WBC 12   Hgb 11   Plt 150  Lytes   Na 140   K 3.9   Cl 110   HCO_3_ 24   AG 10   Urea 20   Cr 0.9   Glucose 150  Extended Lytes   Ca 10   Mg 1.9   PO_4_ 3   Albumin 3.8   TSH wnl  VBG   pH 7.49   pCO_2_ 39   pO_2_ 40   HCO_3_ 22   Lactate 1.7 | Cardiac/Coags   Trop neg x 1   D-dimer wnl   INR wnl   aPTT wnl  Biliary   AST wnl   ALT wnl   GGT wnl   ALP wnl   Bili wnl   Lipase wnl  Tox   EtOH neg   ASA neg   Tylenol neg   Dig level neg   Osmols wnl  Other   B-HCG negative |
| --- | --- |

**Appendix B: ECGs, X-rays, Ultrasounds and Pictures**

| 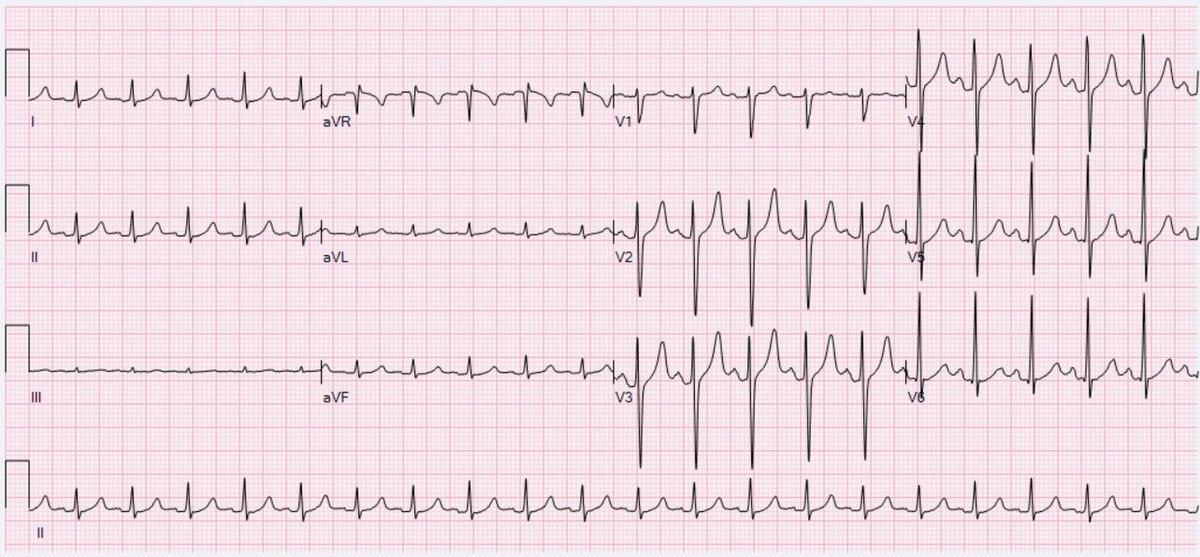  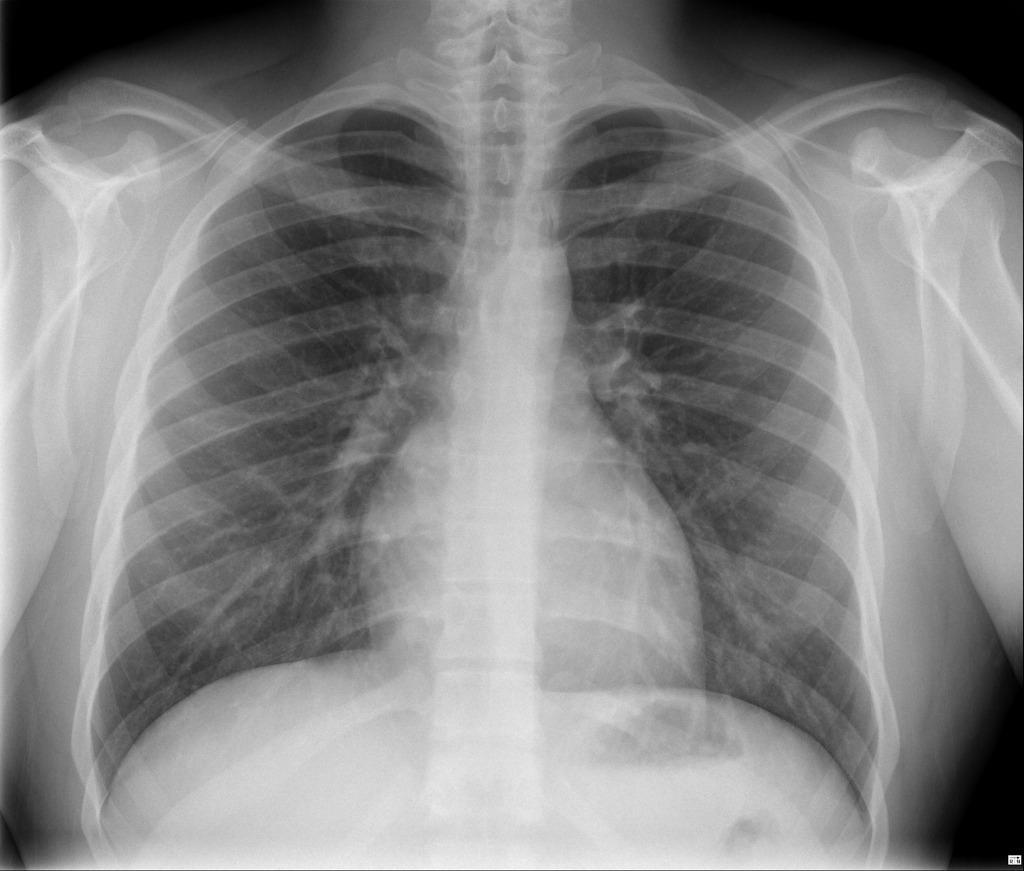    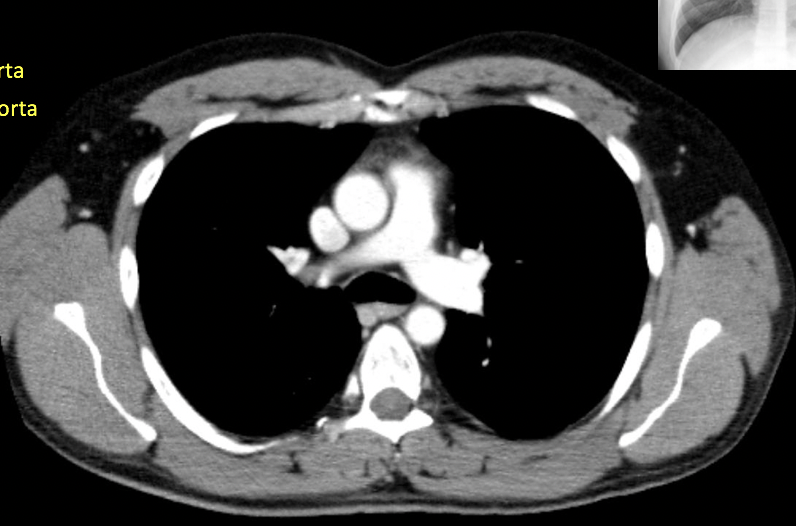  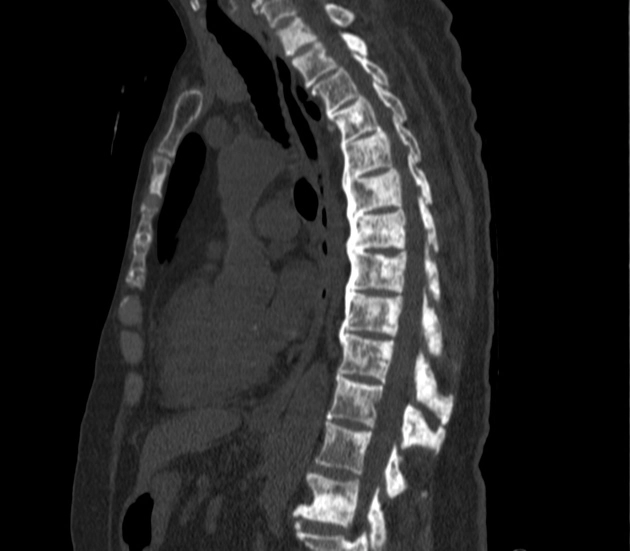 |
| --- |

**SP Case Notes**

**Goals of Care Conversations: Metastatic Spinal Cord Compression**

| **Patient Name and DOB** | **Melissa Grodskaya, 52 yo F**  **SP will play role of patient’s close family member, Jay Wayne Jenkins** |
| --- | --- |
| **Opening Statement/**  **Chief Complaint** | **“My cousin isn’t feeling that well.”** |
| **“Tell Me More About It”** | “I just started supporting her/him through this breast cancer journey, we just started chemo, surgery is up next, but this back pain and high blood pressure was so sudden. I always get her/him to take their medication and on time, every time. How can this?  We’re doing all the things the doctors said to do.” |
| **Demeanor/ Physicality** | ***Concerned but calm, open body language towards providers*** |
| **History of Present Illness** | 52 yo F w/ PMH poorly controlled HTN and recently diagnosed breast cancer here for chest pain, back pain, and left upper and lower extremity weakness and numbness. Patient presents markedly hypertensive, uncomfortable, and appears to initially have an aortic dissection. However, further imaging will reveal she has metastatic disease to the spine, causing her neurologic deficit. Patient will not have an aortic dissection but will show evidence of metastatic disease to the lower cervical/upper thoracic spine. |
| **Past Medical Hx** | Hypertension, Breast Cancer (Diagnosed 3W ago, 1 round of chemotherapy so far) |
| **Past Surgical Hx** | TBD |
| **Allergies** | NKDA |
| **Medications** | Lisinopril 40mg, Amlodipine 10mg, Naproxen 250mg BID |
| **Over the Counter/ Vitamins & Supplements** | **Psyllium Husk 6 capsules daily (3.12g)** |
| **Social Hx** | Teacher for 30 years, no toxic habits |
| **Family Medical Hx** | Unremarkable |
| **ROS/Physical Findings** | **BP: 220/150 HR: 110 SpO2: 98% RR: 26 Temp: 98.7**  **General: Uncomfortable, grimacing, writhing around in pain, mildly diaphoretic**  **Card:** **Tachycardic, regular rhythm, no murmurs, rubs, or**  **Gallops**  **Resp: clear bilateral breath sounds**  **Abd: soft, nontender. Non-distended, no rebound,**  **guarding, rigidity. No CVAT. No pulsatile mass.**  **Head & Neck: supple, no tenderness**  **MSK/Skin: no rash, purpura, or ecchymosis. +Mild left**  **upper thoracic tenderness to palpation. No midline cervical,**  **thoracic, or lumbar spine tenderness to palpation. No step**  **offs.**  **Neuro: CNII-XII intact, PERRL, EOMI**  **3/5 grip strength left hand, 4/5 LUE flexion/extension**  **5/5 right hand, 5/5 RUE, 4/5 LLE dorsiflexion**  **Slow but steady gait, no dysmetria w/ FTN. Normal rapid**  **alternating movements.** |
| **Props/ Moulage** |  |
| **SP Special instructions** | You have noticed that your family member is not only struggling to cope with a recent breast cancer diagnosis and the initial round of chemo plus surgical planning, but they are now having very high blood pressure and weakness.  You and your family member are exacerbated by this next bump in the road.  Initial workup will suggest that there is an acute life-threatening cause of chest and back pain in the setting of high blood pressure, aortic dissection. “I just started supporting her/him through this breast cancer journey, we just started chemo, surgery is up next, but this back pain and high blood pressure was so sudden. I always get her/him to take their medication and on time, every time. How can this? We’re doing all the things the doctors said to do.”  After learning about metastases to the spine which are found on the CT chest-abdomen-pelvis scan: “I just don’t understand, breast cancer and now this? Our family doesn’t deserve this.”  Not realizing metastases means the patient’s breast cancer is terminal:  After realizing that their loved one is much sicker than originally diagnosed: “Melissa has always been so independent in their life, living on their own, traveling the world and things of that nature, will that continue to be the case? Will they be able to live independently still?”  *One of the main objectives of this case is for the team to have a goals of care conversation with you. If they do not prompt you to have this discussion you should bring it up “How likely is it that they make it out of this okay? How much time do we have left?”*  You should be extremely against any thoughts of the patient dying at first, the medical team should be able to give her reassurance that they are focused on symptoms, the medical team should be gentle and caring towards you. Eventually, you will help the patient decide that they want the highest quality of life for the time they have left. |
|  |  |

**Section 1: Case Summary**

| **Scenario Title:** | **End of Life Symptom Management** |
| --- | --- |
| Keywords: | Oncology, pain management, end of life symptom management |
| Brief Description of Case: | 78 yo F w/ recently diagnosed pancreatic cancer who recently received palliative chemotherapy presents for acute generalized weakness, decreased appetite, and dyspnea. Patient will be neutropenic and will require multiple pressors, but will remain hypotensive despite aggressive intervention. Family will later opt for a palliative extubation while in ED. |

| **Goals and Objectives** | |
| --- | --- |
| Educational Goal: | recognize the active dying process, manage symptoms at end of life, perform a palliative extubation |
| Objectives:  (Medical and CRM) | 1. Recognize the active dying process and understand when further medical intervention is futile 2. Manage symptoms at the end of life using appropriate medications 3. Communicate effectively with other team members 4. Communicate bad news effectively to family members 5. Perform palliative extubation |
| EPAs Assessed: |  |

| **Learners, Setting and Personnel** | | | | | |
| --- | --- | --- | --- | --- | --- |
| Target Learners: | ☒ Junior Learners | | ☒ Senior Learners | | ☐ Staff |
|  | ☐ Physicians | ☐ Nurses | | ☐ RTs | ☐ Inter-professional |
|  | ☐ Other Learners: | | | | |
| Location: | ☒ Sim Lab | | ☐ In Situ | | ☐ Other: |
| Recommended Number of Facilitators: | Instructors: 2 | | | | |
|  | Sim Actors:2 | | | | |
|  | Sim Techs: 1 | | | | |

**Section 2A: Initial Patient Information**

| 1. **Patient Chart** | | | | | | |
| --- | --- | --- | --- | --- | --- | --- |
| Patient Name:  Akosua Obduwale | | | | Age: 78 | Gender: F | Weight:64 kg |
| Presenting complaint: Weakness and shortness of breath | | | | | | |
| Temp: 39 C | HR: 115 | BP: 90/50 | | RR: 38 | O_2_Sat: 93% | FiO_2_: 60% |
| Cap glucose: 120 | | | | GCS: (E2 V2 M4 ) 8 | | |
| Sign Out Note:  78 yo F w/ recently diagnosed pancreatic cancer who received palliative chemotherapy 3 days ago presents for acute generalized weakness, confusion, decreased appetite, and dyspnea. Patient initially presented hypotensive, tachycardic, tachypneic with increased work of breathing, altered mental status, and was febrile. Patient was started on peripheral levophed, received 2 L IVF, vancomycin and cefepime, and intubated. Central line was placed and levophed now at 30 mcg/min and has continued to require escalating doses within the past hour. As per daughter and son-in-law on the phone, patient is to remain full code. Patient went into vtach arrest, shocked once, and received 1 amp of calcium, ROSC achieved.  CCM consulted and was rejected to the MICU due to poor prognosis. Family just arrived | | | | | | |
| Allergies: NKDA | | | | | | |
| Past Medical History:  Stage 4 Pancreatic Adenocarcinoma  HTN  HLD | | | Current Medications:  Lisinopril 40 mg daily  Simvastatin 20 mg daily | | | |

**Section 2B: Extra Patient Information**

| **A. Further History** | |
| --- | --- |
| *Include any relevant history not included in triage note above. What information will only be given to learners if they ask? Who will provide this information (mannequin’s voice, sim actors, SP, etc.)?*  Prior to cancer diagnosis a few weeks ago, patient was active, completely independent with all ADLs, and “vibrant.” Patient would travel frequently, visit friends, and cook. While never explicitly addressing code status in the past, patient would often say how she would not want to suffer and hopes she dies at home with family peacefully. | |
| **B. Physical Exam** | |
| *List any pertinent positive and negative findings* | |
| Cardio: Tachycardic, regular, no murmurs | Neuro: Obtunded, withdraws to pain only |
| Resp:  bilateral mechanical breath sounds | Head & Neck: supple, no tenderness |
| Abdo: soft, mild epigastric and RUQ tenderness, +hepatomegaly. no rebound, guarding. | MSK/skin: no rash, purpura, or ecchymosis, diffuse 2+ pitting edema BUE and BLE |
| Other: | |

**Section 3: Technical Requirements/Room Vision**

| **A. Patient** |
| --- |
| ☒ Mannequin *(specify type and whether infant/child/adult): adult* |
| ☐ Standardized Patient |
| ☐ Task Trainer |
| ☐ Hybrid |
| **B. Special Equipment Required** |
| intubation equipment, ventilator, suction |
| **C. Required Medications** |
| RSI medications, vasopressors |
| **D. Moulage** |
| None |
| **E. Monitors at Case Onset** |
| ☐x **Patient on monitor with vitals displayed**  Patient not yet on monitor |
| **F. Patient Reactions and Exam** |
| *Include any relevant physical exam findings that require mannequin programming or cues from patient*  *(e.g. – abnormal breath sounds, moaning when RUQ palpated, etc.) May be helpful to frame in ABCDE format.*  A - intubated on a ventilator  B - adequate ventilation  C - tachycardic, no hemorrhage, weak peripheral pulses  D - obtunded, no sedation, withdraws to pain only  E - no signs of trauma |

**Section 4: Sim Actor and Standardized Patients**

| **Sim Actor and Standardized Patient Roles and Scripts** | |
| --- | --- |
| *Role* | *Description of role, expected behavior, and key moments to intervene/prompt learners. Include any script required (including conveying patient information if patient is unable)* |
| Daughter | Daughter will be visibly upset, intermittently crying, pacing back and forth.  You will be near the patient. If the team does not update you, request that you have an update. Be firm, but not aggressive.  Once you see them try additional resuscitation efforts with no effect, they should warn you that she is actively dying.  If they do not warn you, ask “Is she going to die soon?”  Make note of her grimacing and secretions and say something like “It looks like she’s suffering. She wouldn’t want to go through this. Please no more CPR.”  Request that the patient is extubated.  If they do not attempt to manage her symptoms during the terminal extubation, cry more and start asking the team “Please help her. She looks so uncomfortable!”  If they do not set expectations prior to the extubation, hysterically ask, “What is happening!? Why is she making those noises!? Please make it stop!” |

**Section 5: Scenario Progression**

| **Scenario States, Modifiers and Triggers** | | | | |
| --- | --- | --- | --- | --- |
| Patient State/Vitals | Patient Status | Learner Actions, Modifiers & Triggers to Move to Next State | | Facilitator Notes |
| 1. **Asysolic arrest** | as soon as case starts and team has introduced themselves to the family the patient will code | Expected Learner Actions   - CPR - BVM - Epix1 | Modifiers   - will get ROSC after 1 round | family will be at bedside watching the code and this will push them to make pt DNR and extubate her |
|  |  |  |  |  |
| **2.**  Rhythm: sinus tach  HR: 140  BP: 60/40  RR: 45  O_2_SAT: 90 %  T: 37^o^C  GCS: 3 | Pt grimacing, becoming more tachypneic, overbreathing the vent, increased secretions    Daughter will be crying. Will voluntarily make pt DNR. | Expected Learner Actions  ☐ adjust vent settings  ☐ alert family of worsening clinical status and inform them that death is likely imminent  ☐ have family nearby  ☐ | Modifiers  - Blood pressure will improve for several minutes but will continue to decrease.  - Brief improvement in pulse ox, but will downtrend despite max vent support  -  Triggers  -  - | If family members are nearby, they will not be disruptive. They will just seem sad and anxious. |
| **3. Actively Dying**  Rhythm: sinus  HR: 40  BP: 50/30  RR: 40  O_2_SAT: 60 %  T: 37^o^C  GCS: 3 | Daughter will request that pt be extubated and to be made comfortable.  Pt will continue to grimace, have copious secretions, and have increased work of breathing | Expected Learner Actions  ☐ allow family to be at bedside, provide emotional support  ☐inform family that death will happen very soon  ☐ prepare family for what to expect during and after extubation  ☐ give opioids and/or benzos for dyspnea; titrate up for symptoms  ☐ give atropine for secretions | Modifiers  - if learners do not prepare family, they will become upset and start crying more hysterically  - if learners prepares family and comforts them, they will seem sad but will show gratitude  Triggers  - if extubated, pt will progressively become bradycardic until asystole  - if opioid or benzo dose not increased or titrated up, pt will grimace and appear more uncomfortable (restless, more accessory muscle use) | nurse will have to cue standardized patient about signs of resp distress once extubated. |

**Appendix A: Laboratory Results**

| CBC   WBC 30k   Hgb 10.1   Plt  125  Lytes   Na 131   K 5.9   Cl 111   HCO_3  18_   AG 20   Urea 20   Cr 2.1   Glucose 110  Extended Lytes   Ca 8   Mg 1.6   PO_4  3_   Albumin 3.5   TSH wnl  VBG   pH 7.15 → 7.0   pCO_2  32_   pO_2  40_   HCO_3  20_   Lactate 7→ repeat 15 | Cardiac/Coags   Trop 0.20   D-dimer 0.5   INR wnl   aPTT wnl  Biliary   AST 140   ALT 150   GGT wnl   ALP 120   Bili wnl   Lipase wnl  Tox:   EtOH neg   ASA neg   Tylenol neg   Dig level neg   Osmols neg  Other   B-HCG neg |
| --- | --- |

**SP Case Notes**

**Palliative Extubation Case**

| **Encounter timing (including warning times)** | **10-15 minutes** |
| --- | --- |
| **Post-encounter timing (including warning times)** | **45 minutes** |
| **Feedback details: (i.e. no feedback; timing; faculty & student; SP & student; faculty & SP with student)** | **Faculty, student, SP** |

| **Patient Name and DOB** | **Sarah Obduwale is the 55 year old daughter of  the medical pt** Akosua Obduwale **aged 88 DOB 3/18/35** |
| --- | --- |
| **Opening Statement/**  **Chief Complaint** | **“I'm here because I was told my mother is very sick and may not have much time left.”** |
| **“Tell Me More About It”** | “I just spoke to her yesterday. She had chemo a few days ago but she was in good spirits. And then this morning she just didn’t sound right? Very confused and weak. So I asked a friend to go check on her. Now the doctors are saying she might die?” |
| **Demeanor/ Physicality** | ***Worried, tearful, anxious, pacing*** |
| **History of Present Illness** | Your mother has been in and out of the hospital for the last few weeks. She was diagnosed with pancreatic cancer w. lung metastasis about 8 weeks ago. She has been receiving palliative chemotherapy, last dose was 3 days ago. Today she was acutely weak, confused, short of breath and had loss of appetite. you called a friend to go check on her and the friend found your mother to be ill appearing and called the ambulance. You immediately got into the car to start driving since you live far away. |
| **Past Medical Hx** | Stage 4 Pancreatic Adenocarcinoma  HTN  HLD  preDM |
| **Past Surgical Hx** | None |
| **Allergies** | none |
| **Medications** | Lisinopril 40 mg daily  Simvastatin 20 mg daily  Metformin 1000 mg daily |
| **Over the Counter/ Vitamins & Supplements** | **None** |
| **Social Hx** | ***Smoking: never***  ***Alcohol:socially*** |
| **Family Medical Hx** | ***Parents: blood pressure (dad), diabetes(mom), heart disease(dad)***  ***Siblings: blood pressure (brother)***  ***Children:*** |
| **ROS/Physical Findings** |  |
| **Props/ Moulage** | ***SP should have hand written med list,*** |
| **SP Special instructions** | **You are at the patient’s bedside tearful. You have not yet received an update from the medical team. If the team does not update you, then ask for one. Be firm but not aggressive. You will be very upset at the news of your mother’s grave illness,  but you are even more upset by your mother’s apparent suffering. You and your mother never explicitly discussed her advanced directives but you’ve heard her say on multiple occasions that when her time comes she does not want to suffer. She would rather die at home surrounded by family. She often shared that she’s lived a long and beautiful life and she is ready to meet the Lord.**  **You will watch in horror as you see the team begin resuscitative measures when she loses pulses. While measures are ongoing, they should warn you that she is actively dying. If they do not say anything, you should ask “is she going to die?”**  **Make note of her grimacing and secretions. Share that she is suffering and never would have wanted this. Tearfully request that there be no more CPR should she lose pulses again. Request that the patient be extubated.**  **During the extubation, she will begin to have labored breathing and appear to be in distress. The team should warn you of this. If they do not warn you, you should become hysterical and ask “what is happening?! Why is she making those noises! What have you done to her?! Make it stop!”**  **If they do not attempt to make her symptoms better you should become even more tearful and beg them to help her. “Please! She is suffering even more now!Help her!”** |

**Section 1: Case Summary**

| **Scenario Title:** | **Septic Shock in the demented patient** |
| --- | --- |
| Keywords: | Septic Shock, resuscitation, , palliative care, surrogate decision maker |
| Brief Description of Case: | Older male with poor cognitive status presents in septic shock necessitating pressors/intubation should family want that. |

| **Goals and Objectives** | |
| --- | --- |
| Educational Goal: | Understand and manage legal/ethical issues in regards to surrogate decision makers |
| Objectives:  (Medical and CRM) | 1. Inquire about and manage complexities in surrogate decision makers 2. Understand the order of surrogate decision makers 3. Manage septic shock |
| EPAs Assessed: |  |

| **Learners, Setting and Personnel** | | | | | |
| --- | --- | --- | --- | --- | --- |
| Target Learners: | ☒ Junior Learners | | ☒ Senior Learners | | ☐ Staff |
|  | ☐ Physicians | ☐ Nurses | | ☐ RTs | ☐ Inter-professional |
|  | ☐ Other Learners: | | | | |
| Location: | ☒ Sim Lab | | ☐ In Situ | | ☐ Other: |
| Recommended Number of Facilitators: | Instructors: | | | | |
|  | Sim Actors: 2 | | | | |
|  | Sim Techs: 2 | | | | |

**Section 2A: Initial Patient Information**

| 1. **Patient Chart** | | | | | | |
| --- | --- | --- | --- | --- | --- | --- |
| Patient Name:  Jake F.S. Farm | | | | Age: 63 | Gender: M | Weight:70kg |
| Presenting complaint: AMS | | | | | | |
| Temp: 39C | HR: 135 | BP: 80/40 | | RR: 24 | O_2_Sat: 92% | FiO_2_:RA |
| Cap glucose: 48 | | | | GCS: | | |
| Triage note:  63M BIBEMS from NH for AMS x1day | | | | | | |
| Allergies: penicillin (rash) | | | | | | |
| Past Medical History:  CVA (residual right sided weakness) dementia (baseline ao2, conversant, pleasant)  HTN  HLD  DM | | | Current Medications:  ASA Plavix atorvastatin metoprolol metformin | | | |

**Section 2B: Extra Patient Information**

| **A. Further History** | |
| --- | --- |
| *Include any relevant history not included in triage note above. What information will only be given to learners if they ask? Who will provide this information (mannequin’s voice, sim actors, SP, etc.)?*  63M hx dementia cva htn hld dm presents from NH for AMS. History is obtained from daughter who visits patient regularly and saw patient yesterday at baseline complaining of abd pain today confused and decreased responsiveness. There is no formal HCP, daughter is very involved, son is less involved. Learners will only know of son if they ask about additional family members. | |
| **B. Physical Exam** | |
| *List any pertinent positive and negative findings* | |
| Cardio:  tachycardic | Neuro: moaning, right sided weak from prior stroke not following commands |
| Resp:  bilateral breath sounds | Head & Neck: n/a |
| Abdo: soft, ttp rlq | MSK/skin: warm, clammy |
| Other: | |

**Section 3: Technical Requirements/Room Vision**

| **A. Patient** |
| --- |
| ☒ Mannequin *adult* |
| ☐ Standardized Patient |
| ☐ Task Trainer |
| ☐ Hybrid |
| **B. Special Equipment Required** |
| Intubation equipment, |
| **C. Required Medications** |
| RSI medications, IVF antibiotics |
| **D. Moulage** |
|  |
| **E. Monitors at Case Onset** |
| ☐ Patient on monitor with vitals displayed  ☐ Patient not yet on monitor |
| **F. Patient Reactions and Exam** |
| Pt will be in septic shock from perforated gastric ulcer. Pt will have poor mental status and may require intubation.  A – moaning, audible secretions.  B – tachypnic,  symmetric breaths  C – tachycardic, thready pulses  D – moaning, right side weak at baseline, not following commands  E – no rashes |

**Section 4: Sim Actor and Standardized Patients**

| **Sim Actor and Standardized Patient Roles and Scripts** | |
| --- | --- |
| *Role* | *Description of role, expected behavior, and key moments to intervene/prompt learners. Include any script required (including conveying patient information if patient is unable)* |
| Family member(s) | Daughter: very involved in fathers care visits him in the nursing home everyday. Angry at brother who lives farther away from NH and thus doesn’t visit as much. Sees herself as the medical decision maker BUT there is no health care proxy form signed. Wants everything done for the patient. Will arrive with patient and be the primary history provider for the team. Patient was complaining fo some abd pain yesterday the nursing home said it was gas pain and today pt was confused and vomiting in the nursing home so she demanded they send him to ER. Reports patients baseline as pleasant but confused, needs assistance with most ADLs. Will be very concerned and frantic about father want to know whats wrong want to know what can be done to help father. Will not endorse that her brother is around unless specifically asked.  “what is wrong with my father”  “please do everything to help him”  “hes so strong he is a survivor hes overcome so much”  Son: very caring and realistic about his father but works a lot of hours and doesn’t live as close as his sister and thus doesn’t visit as much. would prefer father to be comfortable and not have invasive  interventions performed on his dad. Will be available in case quickly if team asks initial sibling about other siblings, or will arrive as team is setting up for invasive measures (central line vs intubation) and demand that they stop—want to understand what is going on with dad and realistically how the patient would do with these invasive measures vs without. Will state that dad never wanted to be reliant on machines to live life and is concerned about putting him on a breathing tube will argue with sister about what to do unless team is able to have a calm cool collected conversation about the acuity of the patients condition. Will ultimately agree with sister on aggressive measures given an acute decompensation.  “Dad wouldn’t want to be on a machine for the rest of his life”  “what is the chances that he comes off the tube”  “will he survive if we don’t do these things” |
|  |  |

**Section 5: Scenario Progression**

| **Scenario States, Modifiers and Triggers** | | | | |
| --- | --- | --- | --- | --- |
| Patient State/Vitals | Patient Status | Learner Actions, Modifiers & Triggers to Move to Next State | | Facilitator Notes |
| **1. Baseline State**  HR: 135  BP: 80/40  RR: 24  O_2_SAT:92 %  T: 39^o^C  GCS: | *Pt is moaning, in mild resp distress* | Expected Learner Actions  ☐ place IV, send sepsis labs  ☐ start broad spec abx, ivf  ☐ get history form daughter who is at bedside  ☐ RUSH exam:  ☐ finger stick  ☐  ☐ | Modifiers  *Changes to patient condition based on learner action*  - IVF will minimally improve BP  - mental status will not improve  Triggers  *For progression to next state*  - once pressors are started can move to stage 2  - | Nurse can prompt for labs/finger stick if team does not order |
| **2.**  Rhythm:  HR: 120  BP:100/60  RR: 28  O_2_SAT: 95%  T:39 ^o^C  GCS: | Still moaning still in resp distress but now more hemodynamically stable | Expected Learner Actions  ☐ send pt for CT  ☐  ☐  ☐  ☐ | Modifiers  - once returns from CT resp status will decline and pt will need intubation  -  -  Triggers  -  - | Son will enter the case as team is prepping to intubate. Will get call from radiology that pt has a perforated gastric ulcer as prepping to intubate. Will need to discuss with family what is going on and guide through a family discussion on interventions as son does not want invasive measures but will give in once explained what is happening. |
| **3.**  HR: 120  BP:110/70  RR: vent  O_2_SAT: 100%  T:39 ^o^C  GCS: | intubated | Expected Learner Actions  ☐ call surgery for disposition  ☐  ☐  ☐  ☐ | Modifiers  -  -  -  Triggers  -  - |  |
| **4.** |  | Expected Learner Actions  ☐  ☐  ☐  ☐  ☐ | Modifiers  -  -  -  Triggers  -  - |  |

**Appendix A: Laboratory Results**

| CBC   WBC 27   Hgb  11   Plt 400  Lytes   Na    127   K       5.2   Cl       100   HCO_3_ 14   AG     20   Urea   50   Cr     1.5   Glucose    48  VBG   pH        7.22   pCO_2_ 26   pO_2_   HCO_3_ 11   Lactate    5 | Cardiac/Coags   Trop             0.33  INR              1.1    Biliary   AST          45   ALT          48   Bili            1   Lipase     <20  Tox   EtOH   ASA   Tylenol   Dig level   Osmols |
| --- | --- |

**Appendix B: ECGs, X-rays, Ultrasounds and Pictures**

| 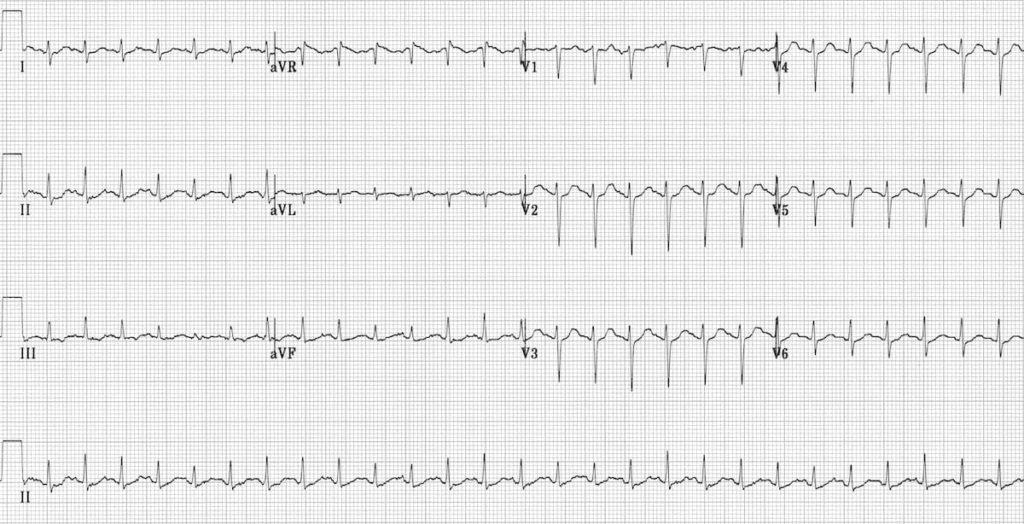    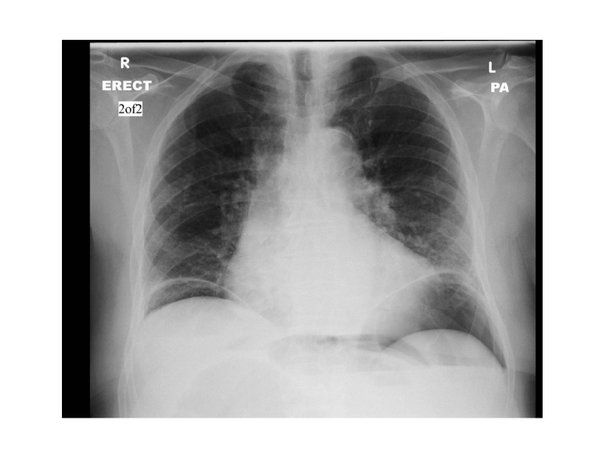 |
| --- |

**SP Case Notes**

**Kelly Farm – Advanced Directives**

| **Patient Name and DOB** | **Kelly Farm is the 35 year old daughter of  the medical pt Jake Farm aged 63** |
| --- | --- |
| **Opening Statement/**  **Chief Complaint** | **“My dad hasn’t been himself the last day, something is wrong”** |
| **“Tell Me More About It”** | “He has been in the nursing home since his stroke, I visit him every day after work. Yesterday I went to see him and he was complaining of some stomach pains above his belly button and then I got a call from the nursing home today that he seemed confused. I told them to send him to the hospital and I would meet himhere. Hes normally so lively talking nonstop this just isn’t him. He looks like he is in pain.” |
| **Demeanor/ Physicality** | ***Worried, nervous, anxious, pacing*** |
| **History of Present Illness** | Your father suffered a severe stroke one year ago which cause right sided weakness and caused him to go into a nursing home. He has had such a positive outlook on everything and makes the most of every day. You visit him everyday, having dinner, watching tv, playing games. On and off the last few days he had seemed uncomfortable after dinner but he never complains so you let it be. Yesterday he started complaining of the pain and you got worried but the nurses assured you everything was ok. Dad didn’t eat his whole dinner which worried you as well but he had no fevers no nausea or vomiting. Today while getting ready to go to work you received a call from the NH that your dad was more sleepy that usual and seemed confused and had been vomiting. You asked to speak to him and he sounded unwell and you asked thqt they send him  to the hospital |
| **Past Medical Hx** | ***Stroke last year- residual right sided weakness***  ***Dementia: diagnosed four years ago, baseline ao2, conversant pleasant***  ***High blood pressure: diagnosed 20 years ago***  ***High cholesterol: diagnosed 20 yaers ago***  ***Diabetes: Diagnosed 10 years ago*** |
| **Past Surgical Hx** | None |
| **Allergies** | none |
| **Medications** | ***Metoprolol 25mg (blood pressure) in AM, PM***  ***Aspirin 81mg (stroke prevention) in AM***  ***Plavix75mg  (stroke prevention) in AM***  ***atorvastatin (cholesterol)in AM***  ***metformin (diabetes)in AM*** |
| **Over the Counter/ Vitamins & Supplements** | ***multivitamin*** |
| **Social Hx** | ***Smoking:10 pack year smoking history***  ***Alcohol:socially*** |
| **Family Medical Hx** | ***Parents: blood pressure (dad), diabetes(mom), heart disease(dad)***  ***Siblings: blood pressure (brother)***  ***Children:*** |
| **ROS/Physical Findings** |  |
| **Props/ Moulage** | ***SP should have hand written med list,*** |
| **SP Special instructions** | ***You are very involved in your fathers care and you visit him every day at the nursing home. You consider yourself his sole caretaker – your mother passed away some time ago and your brother lives farther away and has less flexibility in his job and doesn’t come visit as much. You resent your brother and are angry that he isn’t as involved. You see yourself as the medical decision maker BUT THERE IS NO HEALTH CARE PROXY FORM SIGNED (which means any advanced care directives would need to be agreed upon by you and your sibling)***  ***You will want everything to be done for your dad, he has been so strong since his stroke and you think he can survive anything. You will be the primary history provider as your dad is confused and will be unable to speak for himself.***  ***If asked specific questions about your dads functional status: he is normally conversant, happy, but needs assistance with bathing, dressing, eating, preparing food and most other activities of daily living.***  ***You will be very concerned and frantic about your dad and what to know whats wrong  with him and know what needs to be done to make him better. YOU WILL NOT MENTION YOUR BROTHER UNLESS SPECIFICALLY ASKED***  ***“what is wrong with my dad?”***  ***“please do everything to help him”***  ***“he is so strong, he has overcome so much already, you have to do whatever needs to be done”***  ***At some point in the case your brother will show up and he will not want invasive measures performed on your dad. At first you will be angry with him “you never cared about dad now you just want to let him die”He is coming from a good place of not wanting dad to suffer and you will soften if he is given an opportunity to explain himself. “Dad never wantd to be dependent on machines to live” you will come together and start asking thoughtful questions about how likely it is that dad would be reliant on machines if invasive measures were done. “how likely is he to survive with vs without these interventions” and eventually you will agree together to proceed with central line/intubation/surgery*** |

**SP Case Notes**

**Brandon  Farm – Advanced Directives**

| **Patient Name and DOB** | **Brandon Farm is the 31 year old son of  the medical pt Jake Farm aged 63** |
| --- | --- |
| **Opening Statement/**  **Chief Complaint** | **“n/a”** |
| **“Tell Me More About It”** | n/a |
| **Demeanor/ Physicality** | ***Frustrated, logical, realistic,*** |
| **History of Present Illness** |  |
| **Past Medical Hx** | ***Stroke last year- residual right sided weakness***  ***Dementia: diagnosed four years ago, baseline ao2, conversant pleasant***  ***High blood pressure: diagnosed 20 years ago***  ***High cholesterol: diagnosed 20 yaers ago***  ***Diabetes: Diagnosed 10 years ago*** |
| **Past Surgical Hx** | None |
| **Allergies** | none |
| **Medications** | ***Metoprolol 25mg (blood pressure) in AM, PM***  ***Aspirin 81mg (stroke prevention) in AM***  ***Plavix75mg  (stroke prevention) in AM***  ***atorvastatin (cholesterol)in AM***  ***metformin (diabetes)in AM*** |
| **Over the Counter/ Vitamins & Supplements** | ***multivitamin*** |
| **Social Hx** | ***Smoking:10 pack year smoking history***  ***Alcohol:socially*** |
| **Family Medical Hx** | ***Parents: blood pressure (dad), diabetes(mom), heart disease(dad)***  ***Siblings: blood pressure (brother)***  ***Children:*** |
| **ROS/Physical Findings** |  |
| **Props/ Moulage** | ***SP should have hand written med list,*** |
| **SP Special instructions** | ***You will arrive partway through the case. Either you will show up shortly after the team asks about additional family and your sister mentions you, or just as the team is setting up for an invasive procedure (central line or intubation)***  ***If you arrive as team is preparing for invasive measures you will be more angry than if your sister mentions you.***  ***“Heywhaty are you doing to my dad. Stop”***  ***As your sister yells at you you will remain calmer and not rise to her level—you will stop her and want to understand firt what is going on with your dad and understand what would happen to him without invasive measures vs with them because your dad had relayed to you that he would not want to be reliant on machines for the rest of his life and you are worried that if you proceed with invasive measures that would be the outcome.***  ***“what are the chances he could come off the breathing machine”***  ***“how likely is he to survive if we don’t do these things”***  ***eventually you will agree together to proceed with central line/intubation/surgery IF AND ONLY IF the team is able to calm your sister down and lead a thoughtful and clear family meeting in the moment*** |

**Section 1: Case Summary**

| **Scenario Title:** | **Necrotizing Pancratitis- Pain Management** |
| --- | --- |
| Keywords: | Palliative care, pain management, pancreatitis, abdominal compartment syndrome, ARDS |
| Brief Description of Case: | 60 yo F w/ PMH ETOH abuse, alcoholic cirrhosis, HTN, DM here for 2 days of severe diffuse abdominal pain, nausea/vomiting, and generalized weakness. Pt develops necrotizing pancreatitis, becomes hypotensive despite fluids resuscitation. Pt also develops ARDS as well as abdominal compartment syndrome. |

| **Goals and Objectives** | |
| --- | --- |
| Educational Goal: |  |
| Objectives:  (Medical and CRM) | 1. Identify pancreatitis and its various etiologies 2. Appropriately resuscitate patients 3. Recognize signs and symptoms of abdominal compartment syndrome 4. Provide appropriate and adequate pain management |
| EPAs Assessed: |  |

| **Learners, Setting and Personnel** | | | | | |
| --- | --- | --- | --- | --- | --- |
| Target Learners: | ☒ Junior Learners | | ☒ Senior Learners | | ☐ Staff |
|  | ☐ Physicians | ☐ Nurses | | ☐ RTs | ☐ Inter-professional |
|  | ☐ Other Learners: | | | | |
| Location: | ☒ Sim Lab | | ☐ In Situ | | ☐ Other: |
| Recommended Number of Facilitators: | Instructors: 2 | | | | |
|  | Sim Actors:2 | | | | |
|  | Sim Techs: 1 | | | | |

**Section 2A: Initial Patient Information**

| 1. **Patient Chart** | | | | | | |
| --- | --- | --- | --- | --- | --- | --- |
| Patient Name:  Geraldina Ramos | | | | Age: 60 | Gender: F | Weight: 75kg |
| Presenting complaint: Weakness, abdominal pain, nausea/vomiting | | | | | | |
| Temp: 99.9F | HR: 120 | BP: 148/90 | | RR: 20 | O_2_Sat: 95% | FiO_2_:RA |
| Cap glucose: 130 | | | | GCS: (E V M ) 15 | | |
| Sign out  60 yo F w/ PMH EtOH abuse (last drink 4 hours ago), cirrhosis, HCC, DM, HTN and no PSH came to ED about 6 or so hours ago for 2 days of severe abdominal pain, nausea/vomiting (NBNB). not relieved with home pain medications . Lipase is very elevated. Has received 3L IVF so far and morphine 4 mg IV x 2 with improvement in pain. Just received CT scan. Follow up the read. She looks fine, maybe a little tachy, but could probably go to floors. I would keep going giving her more fluids. Her LV function looks fine. If she does get worse, may need CCM consult for dispo. | | | | | | |
| Allergies: Ibuprofen | | | | | | |
| Past Medical History:  ETOH abuse HCC  Cirrhosis  DM  HTN  pancreatitis | | | Current Medications:  Losartan 100 mg  Metformin 1000 mg BID  morphine ER 90mg BID | | | |

**Section 2B: Extra Patient Information**

| **A. Further History** | |
| --- | --- |
| *Include any relevant history not included in triage note above. What information will only be given to learners if they ask? Who will provide this information (mannequin’s voice, sim actors, SP, etc.)?*  SP at bedside as history giver and advocate. | |
| **B. Physical Exam** | |
| *List any pertinent positive and negative findings* | |
| Cardio: Tachycardic, regular rhythm. No M/R/G. | Neuro: A+Ox3, follows commands, grossly non-focal |
| Resp:  bilateral breath sounds; slightly diminished at bases | Head & Neck: supple, no tenderness |
| Abdo: Very firm, diffusely tender especially over epigastric region. No rebound, guarding. No CVAT. | MSK/skin: no rash, purpura, or ecchymosis  2+ BUE and BLE pitting edema |
| Other: | |

**Section 3: Technical Requirements/Room Vision**

| **A. Patient** |
| --- |
| ☒ Mannequin *(specify type and whether infant/child/adult): adult* |
| ☐ Standardized Patient |
| ☐ Task Trainer |
| ☐ Hybrid |
| **B. Special Equipment Required** |
| Intubation equipment, ventilator |
| **C. Required Medications** |
| RSI medications, vasopressors, ACLS drugs |
| **D. Moulage** |
| None |
| **E. Monitors at Case Onset** |
| ☐ Patient on monitor with vitals displayed  ☒ Patient not yet on monitor |
| **F. Patient Reactions and Exam** |
| *Include any relevant physical exam findings that require mannequin programming or cues from patient*  *(e.g. – abnormal breath sounds, moaning when RUQ palpated, etc.) May be helpful to frame in ABCDE format.*  Airway – protecting airway  Breathing – bilateral breath sounds 🡪 eventually develops crackles  Circulation – tachycardic🡪 worsens and becomes hypotensive  Disability – initially A+Ox3 🡪 becomes altered  Exposure – no ecchymoses or signs of trauma |

**Section 4: Sim Actor and Standardized Patients**

| **Sim Actor and Standardized Patient Roles and Scripts** | |
| --- | --- |
| *Role* | *Description of role, expected behavior, and key moments to intervene/prompt learners. Include any script required (including conveying patient information if patient is unable)* |
| Family member:  Son | Son will call for an update. ***Entire conversation will occur via phone***  “What is going on with my mother?”  “Why is surgery going to see her?”  ***If intubation or GOC are brought up:***  “I don’t think she would want that! She said she would rather die than be placed on a machine. But I’m not sure what to do.”  “Will she ever come off the vent?”  “Being able to spend time with her family and friends was super important for her. She loved to sing and dance bachata. If she couldn’t do those things, I don’t think she would feel like life is worth living.”  ***If no GOC discussion held****:*  Son will be upset. Crying, begging to be allowed in.  He will ask questions about details of her care, prognosis, treatment, etc. |

**Section 5: Scenario Progression**

| **Scenario States, Modifiers and Triggers** | | | | |
| --- | --- | --- | --- | --- |
| Patient State/Vitals | Patient Status | Learner Actions, Modifiers & Triggers to Move to Next State | | Facilitator Notes |
| **1. Baseline State**  Rhythm: sinus tachycardia  HR: 120  BP: 148/90  RR: 22  O_2_SAT:98 %  T: 37^o^C  GCS: 15 | Uncomfortable, complaining of persistent abdominal pain and nausea | Expected Learner Actions  ☐ Give additional analgesics and antiemetics  ☐ Give additional IVF  ☐ Repeat abdominal exam  ☐ Consult general surgery  ☐ Start heparin gtt  ☐  ☐ | Modifiers  *Changes to patient condition based on learner action*  -  -  Triggers  *For progression to next state*  - RN will say warn team that pt made 150 cc’s of urine  - Radiology will call for prelim read of CT scan | Radiology will call and report necrotizing pancreatitis and SMV occlusive thrombus.  General surgery will evaluate patient and say not concerned for abdominal compartment syndrome. Does say to board pt to SICU, but no beds available. |
| **2.**  Rhythm: sinus tachycardia  HR: 145  BP: 150/94  RR:35  O_2_SAT: 92%  T: 37^o^C  GCS: 15 | Complaining of worsening shortness of breath and worsening abdominal pain and distension | Expected Learner Actions  ☐ Obtain repeat CXR, consider intubation  ☐ Place supplemental O2  ☐ Pain management  ☐ Update general surgery consult  ☐ Discuss GOC with patient and son | Modifiers  - If gives more IVF, pt will become more tachypneic and hypoxic  -  -  Triggers  - RN will be concerned about worsening abdominal distension  - Son will call and demand an update* see script above |  |
| **3.**  Rhythm: sinus tachycardia  HR: 162  BP: 88/62  RR: 42  O_2_SAT: 82 %  T: 37^o^C  GCS: 11 (E3V3M5) | Mental status worsens and more tachypneic.  Abdominal distension significantly worse. | Expected Learner Actions  ☐ Start vasopressors  ☐ Intubate patient  ☐ Adjust vent settings for ARDS  ☐ Start appropriate sedation after intubation  ☐ Call general surgery stat to update them if not already done  ☐ Obtain CCM consult for dispo | Modifiers  - If pt is intubated prior to starting pressors, pt will code  Triggers  - After intubation, surgery will come back to check on patient.  - *If time allows, if pt not adequately sedated, pt will buck the vent, become more hypotensive and code. | General surgery will eventually return and perform bedside ex-lap on pt. BP will improve and pressor requirement will decrease. Case will end after ex-lap. |

**Appendix A: Laboratory Results**

***See attached images below for pertinent labs.*

| CBC   WBC   Hgb   Plt  Lytes   Na   K   Cl   HCO_3_   AG   Urea   Cr   Glucose  Extended Lytes   Ca   Mg   PO_4_   Albumin   TSH  VBG   pH   pCO_2_   pO_2_   HCO_3_   Lactate | Cardiac/Coags   Trop   D-dimer   INR   aPTT  Biliary   AST   ALT   GGT   ALP   Bili   Lipase  Tox   EtOH   ASA   Tylenol   Dig level   Osmols  Other   B-HCG |
| --- | --- |

**Appendix B: ECGs, X-rays, Ultrasounds and Pictures**

| 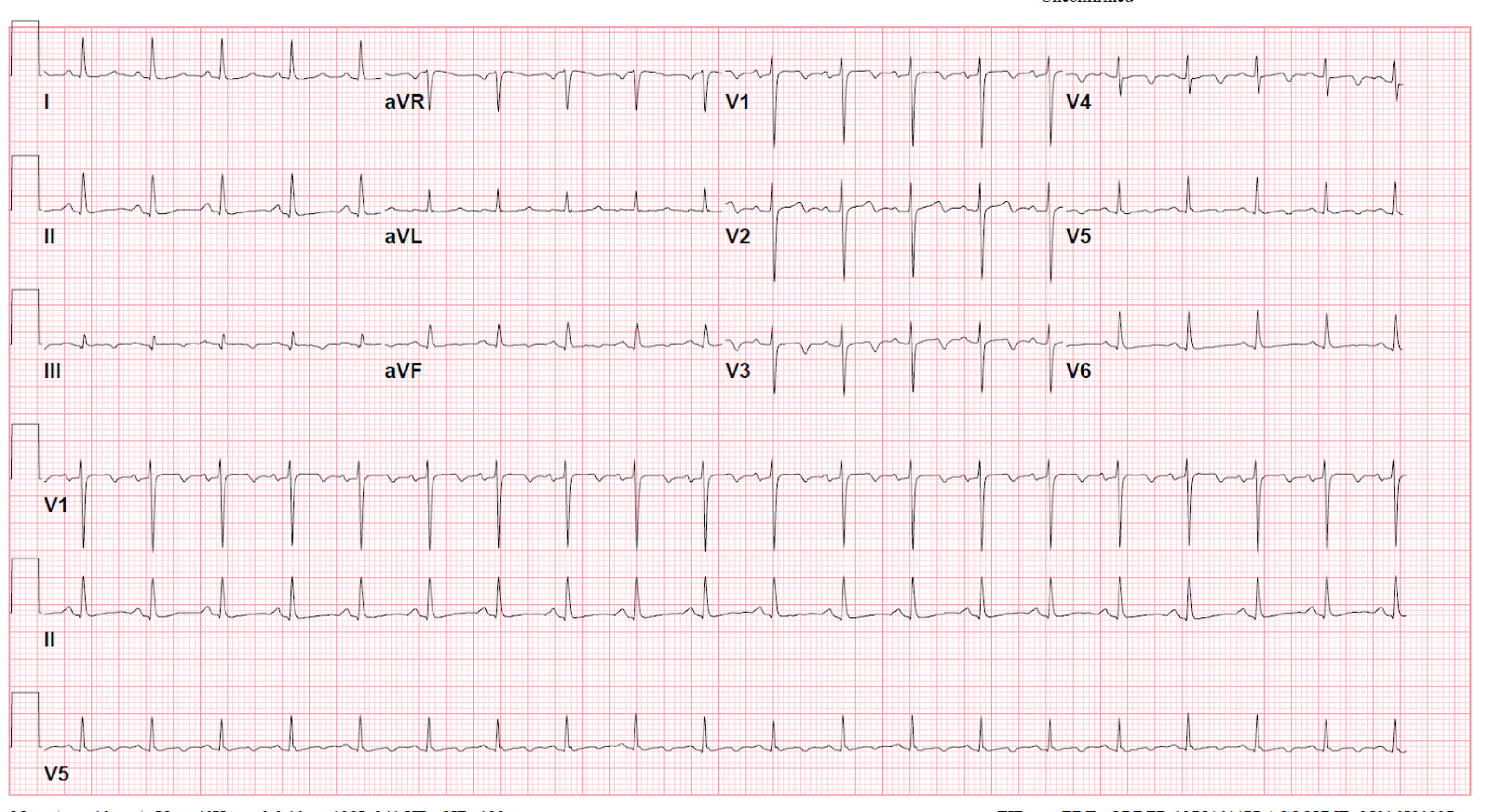    **FIRST CXR**  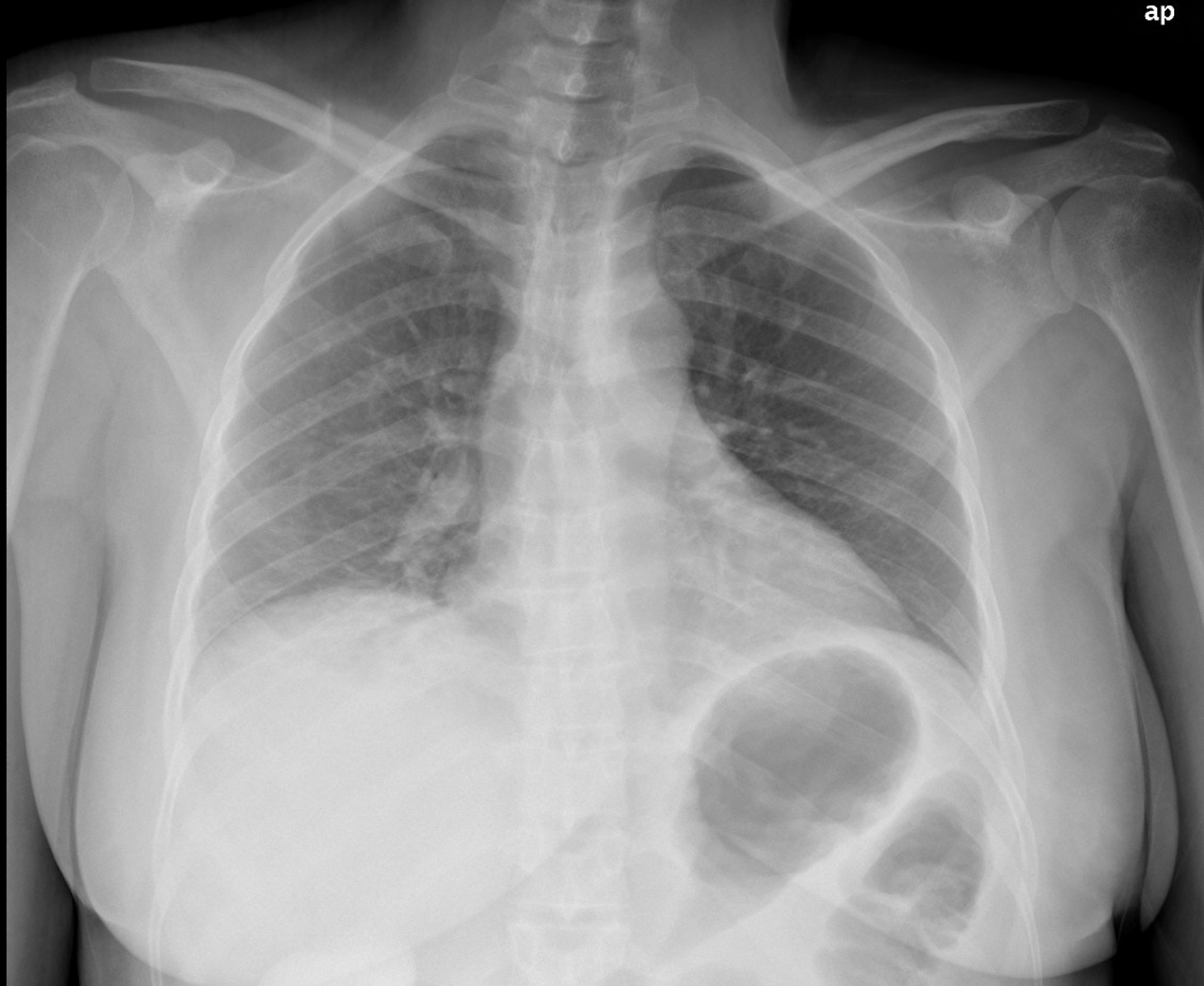    **SECOND CXR**  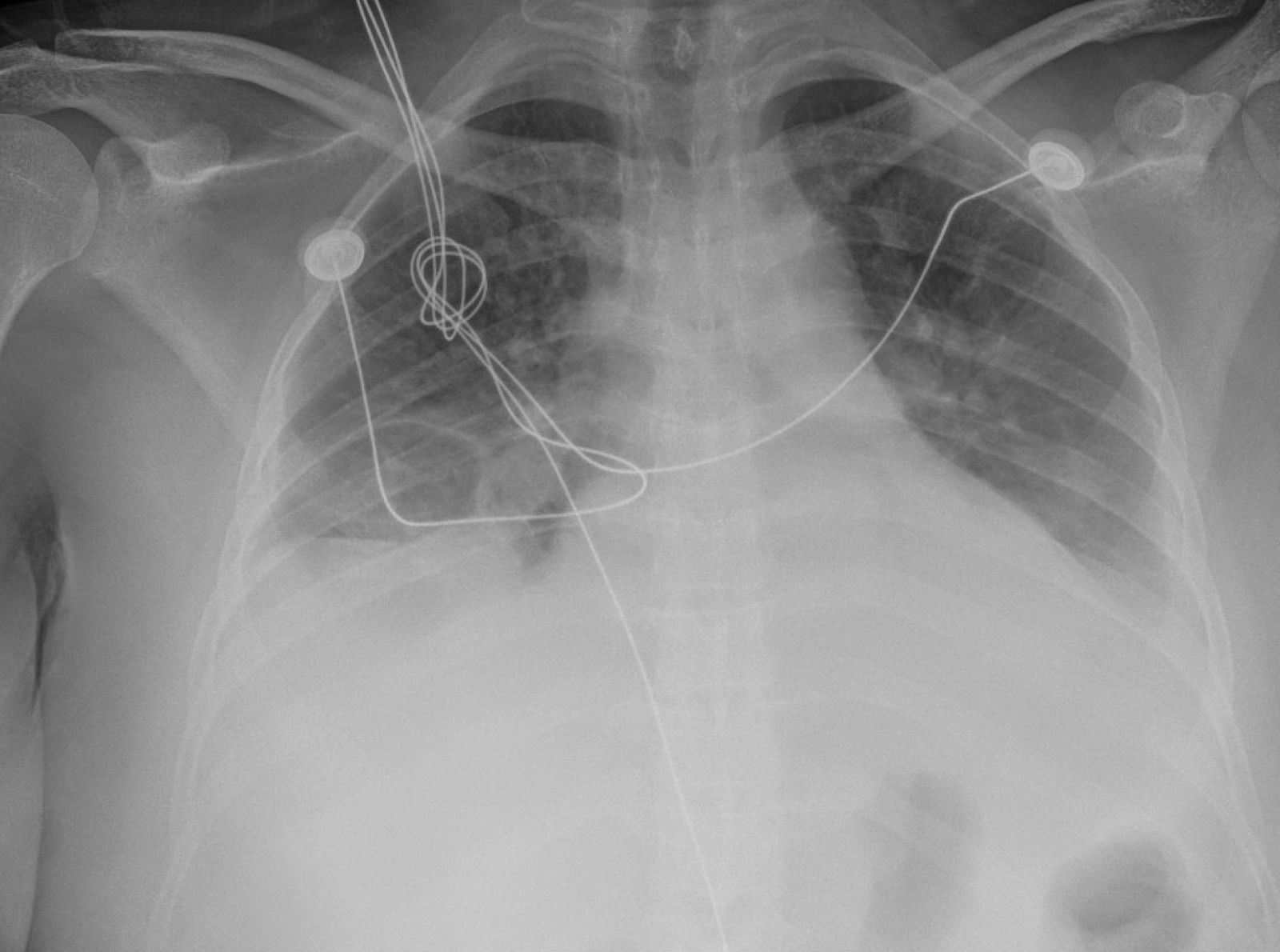    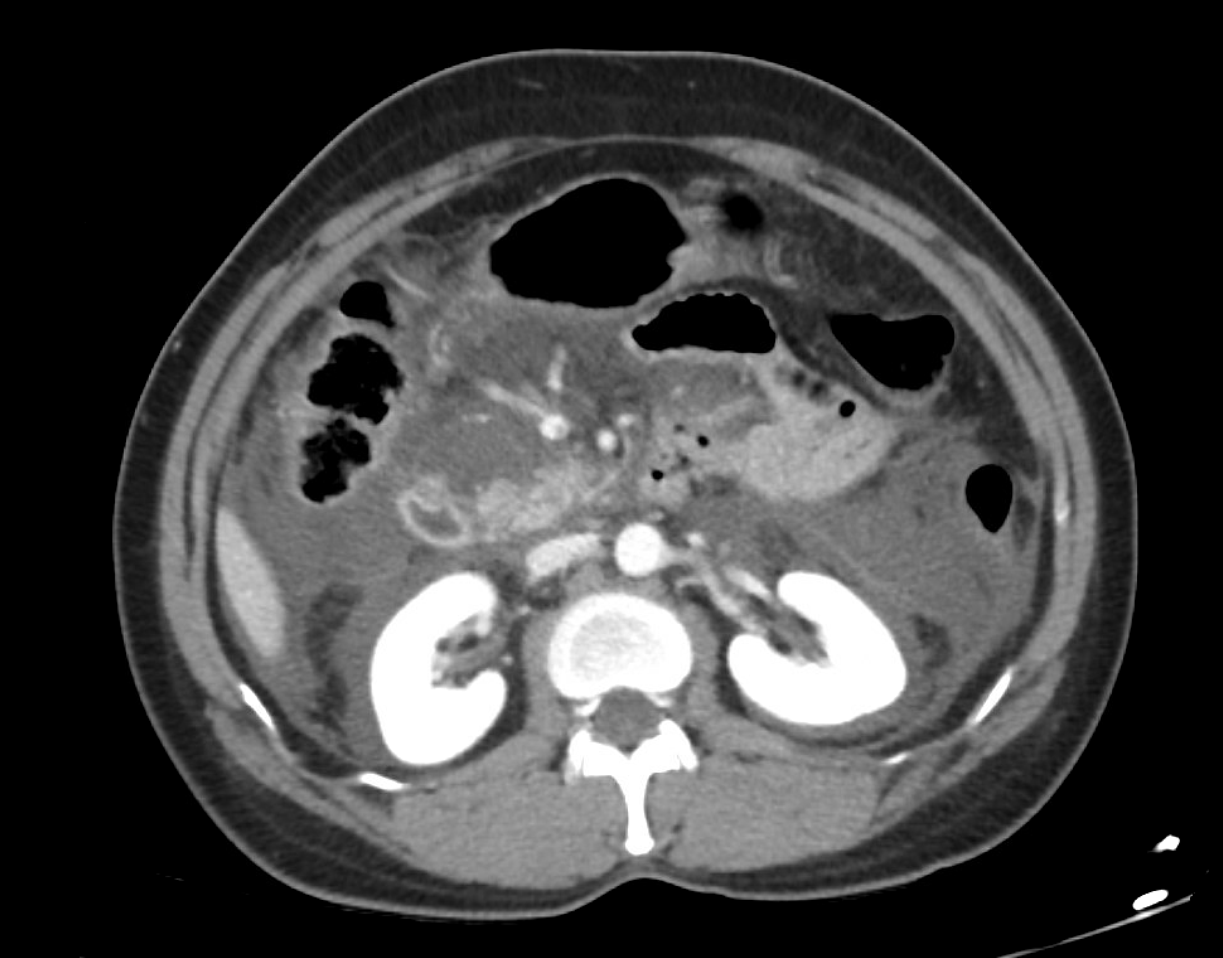  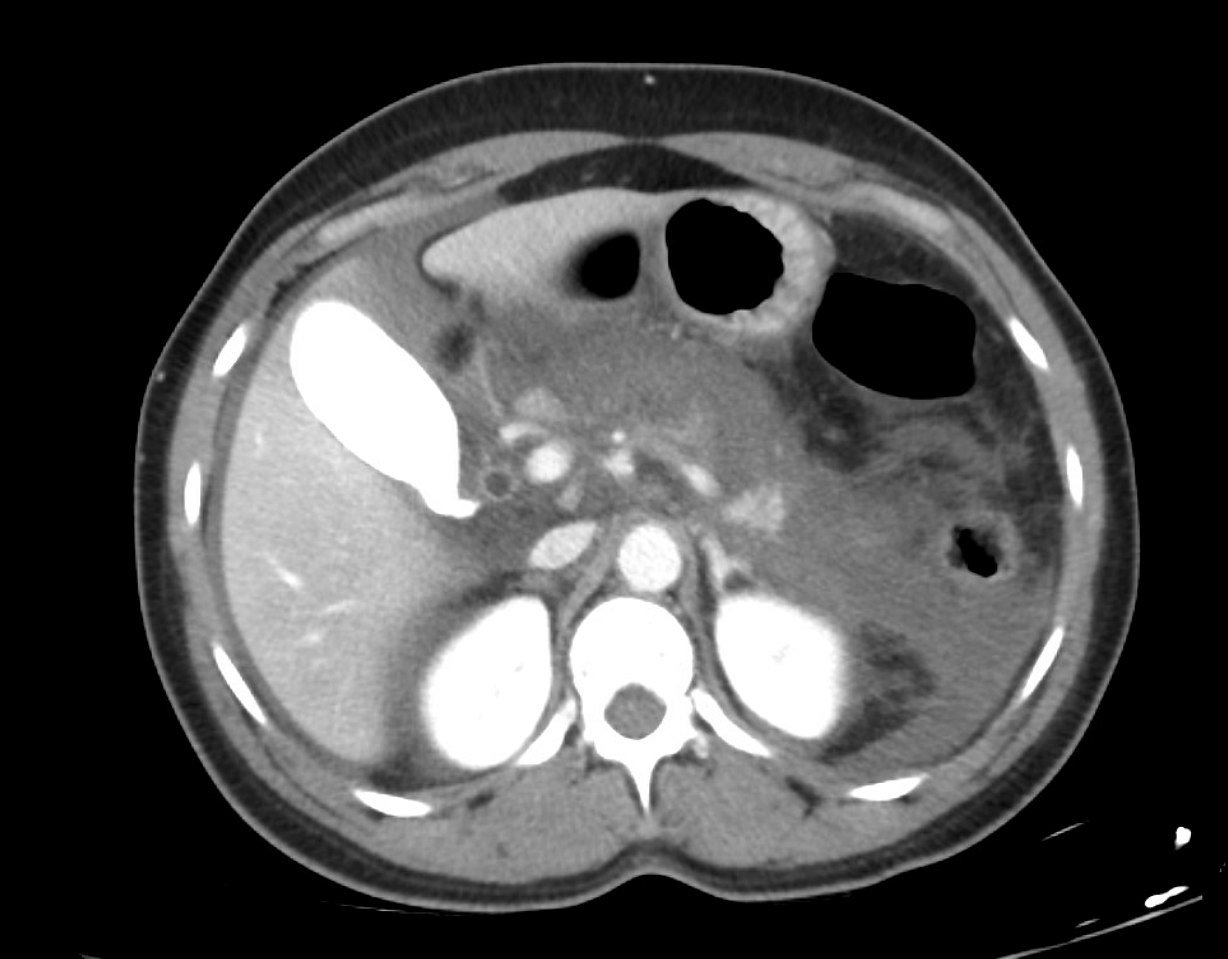  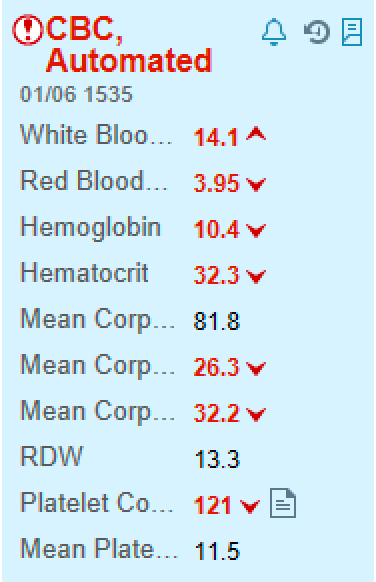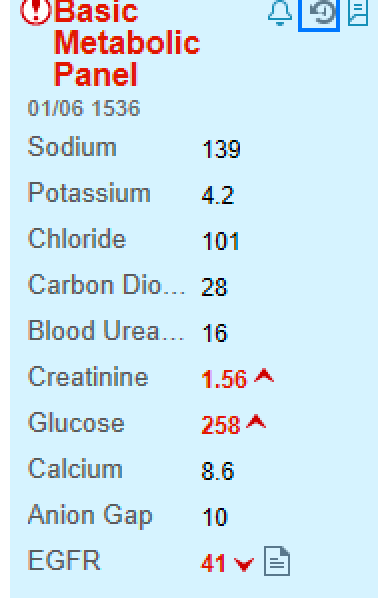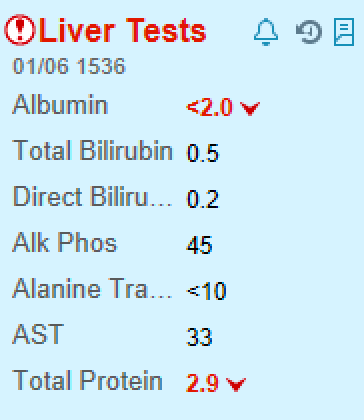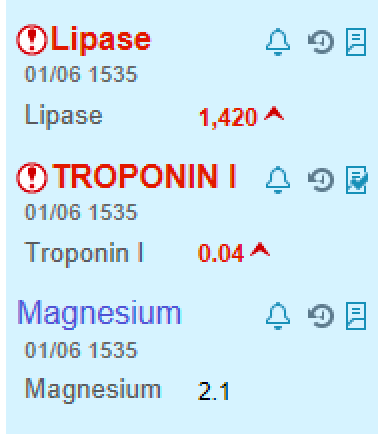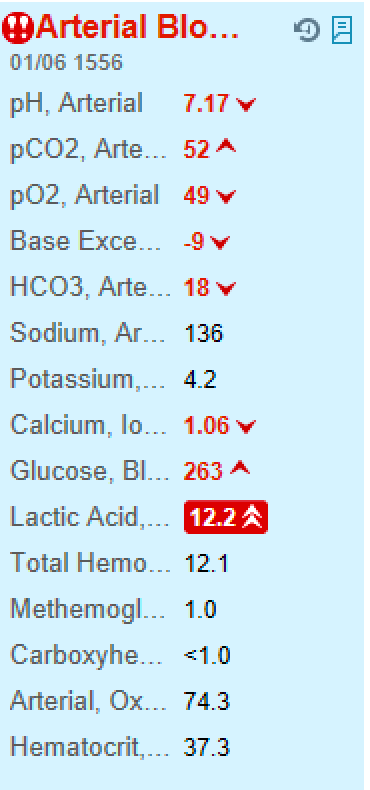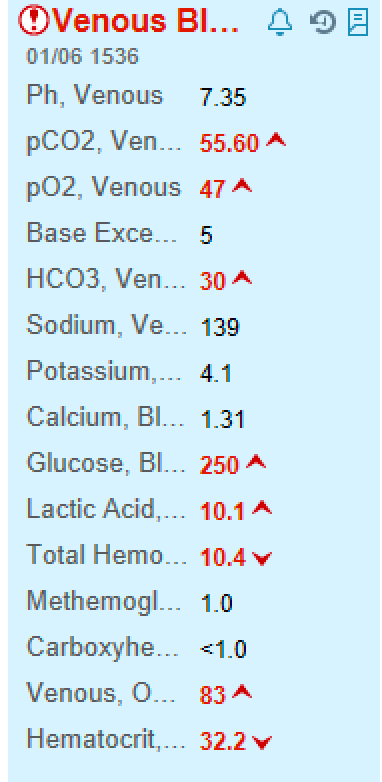 |
| --- |

**SP Case**

**Pain management**

| **Patient Name and DOB** | Daniel Ramos is the 32 year old son of 60 year old patient Geraldina Ramos 4/24/63 |
| --- | --- |
| **Opening Statement/**  **Chief Complaint** | **“I'm here because I was told my mother is very sick and I want to make sure her pain is taken seriously.** |
| **“Tell Me More About It”** | “I know my mom has a drinking problem and it’s made her liver sick and now she has cancer. But she’s a really sweet lady who minimizes her pain so I have to advocate for her” |
| **Demeanor/ Physicality** | ***anxious , frustrated but redirectable*** |
| **History of Present Illness** | Your mother has been in and out of the hospital for the last few weeks. She was diagnosed with liver cancer with bone metastasis several months ago. She takes a lot of opiates at home for pain control. She still struggles with her drinking sometimes. She had a few drinks yesterday when the pain became overwhelming and began vomiting more and complaining of worsening abdominal pain overnight. You called the ambulance and came right away |
| **Past Medical Hx** | Alcohol abuse  Cirrhosis with progression to hepatocellular carcinoma with bone metastasis  Recurrent pancreatitis  High blood pressure  Diabetes |
| **Past Surgical Hx** | None |
| **Allergies** | none |
| **Medications** | Losartan 100 mg daily (for blood pressure)  Metformin 1000 mg BID  (for diabetes)  Morphine 90mg BID (for cancer pain) |
| **Over the Counter/ Vitamins & Supplements** | **None** |
| **Social Hx** | ***Smoking: never***  ***Alcohol: almost daily drinker*** |
| **Family Medical Hx** | ***Parents: blood pressure (dad), diabetes(mom), heart disease(dad)***  ***Siblings: blood pressure (brother)***  ***Children:*** |
| **ROS/Physical Findings** |  |
| **Props/ Moulage** | ***SP should have hand written med list,*** |
| **SP Special instructions** | **You are at the patient’s bedside upset. You have not yet received an update from the medical team and your mom’s pain is getting worse. If the team does not update you, then ask for one. Be sharp but not aggressive. You will be very upset at your mother’s pain not being addressed and irritated that you don’t know her disposition (admit versus discharge).**  **You don’t understand why she needs to see the surgeons or critical care teams. She has had pancreatitis many times and she has never been this sick. You make note that her stomach is much more distended than it was when you came in.**  **You become very concerned as her pain worsens and seems to have trouble breathing. You never explicitly discussed her advanced directives but you’ve heard her say that she would rather die than be placed on a machine. “Being able to spend time with her family and friends was super important for her. She loved to sing and dance bachata. If she couldn’t do those things, I don’t think she would feel like life is worth living. But I don’t know. We thought she had more time so we never talked seriously about this”**  **You will watch in horror as you see her pain and vital signs worsen. You are worried about the level of pain that she is in and if she will make it through this illness. You should become panicked when they make mention that she may need to be intubated. If you are not addressed and no goals of care discussion is had, you should become angry and demand to be spoken with.**  **After the intubation, you are very concerned about her prognosis, next steps of her care, and her level of suffering. You are worried that intubation may not have been the right choice for her. You make note of her abdominal distension and also her grimacing in pain.** |
| **Door Chart** | PATIENT NAME:  Geraldina Ramos  DOB: 4/24/63  Rhythm: sinus tach  HR: 120  BP: 148/90  RR: 20  O_2_SAT: 95 %  T: 99.9^o^F  GCS: 15  60 yo F w/ PMH EtOH abuse (last drink 4 hours ago), cirrhosis, c/b HCC w/ mets to bone, DM, HTN and no PSH came to ED about 6 or so hours ago for 2 days of severe abdominal pain, nausea/vomiting (NBNB). Lipase is very elevated. Has received 3L IVF so far and morphine 4 mg IV x 2 with improvement in pain. Just received CT scan. Follow up the read. She looks fine, maybe a little tachy, but could probably go to floors. I would keep going giving her more fluids. Her LV function looks fine. If she does get worse, may need CCM consult for dispo.  You are to:   1. Stabilize the patient 2. Discuss goals of care with the son |

**Section 1: Case Summary**

| **Scenario Title:** | **Uncontrolled Cancer Pain/  Hospice Care** |
| --- | --- |
| Keywords: | Oncology, pain management, chronic pain, fall, hospice |
| Brief Description of Case: | 55-year-old female with a history of active breast cancer with bone mets, nephrolithiasis. Her breast cancer and metastases have been progressively worsening despite treatment, and she recently moved to hospice where she is on regular PRN opioid pain control. Pt is very short of breath and cannot provide much additional history. Hospice staff note she had a mechanical witnessed fall two days ago and has been experiencing worsening right sided chest and flank pain and shortness of breath since. Her usual PRN oral opiate regimen as well as ibuprofen have not provided her relief. |

| **Goals and Objectives** | |
| --- | --- |
| Educational Goal: | Diagnose life-threatening intrathoracic pathology, treat pain effectively, GOC discussions |
| Objectives:  (Medical and CRM) | 1. Create wide differential diagnosis in acute flank, chest pain, shortness of breath 2. Select medication class that matches a patient’s pain type and pattern 3. Recognize potential adverse effects of opioid therapy 4. Effectively communicate with family member of an incapacitated patient in order to ascertain medical hx, allergies, and code status 5. Discuss goals of care, consent, risks and benefits of emergent treatment and procedures |
| EPAs Assessed: |  |

| **Learners, Setting and Personnel** | | | | | |
| --- | --- | --- | --- | --- | --- |
| Target Learners: | ☒ Junior Learners | | ☒ Senior Learners | | ☐ Staff |
|  | ☐ Physicians | ☐ Nurses | | ☐ RTs | ☐ Inter-professional |
|  | ☐ Other Learners: | | | | |
| Location: | ☒ Sim Lab | | ☐ In Situ | | ☐ Other: |
| Recommended Number of Facilitators: | Instructors: 2 | | | | |
|  | Sim Actors: 1 | | | | |
|  | Sim Techs: 1 | | | | |

**Section 2A: Initial Patient Information**

| 1. **Patient Chart** | | | | | | |
| --- | --- | --- | --- | --- | --- | --- |
| Patient Name:   Melissa Wilson | | | | Age: 55 | Gender:  F | Weight: 75 kg |
| Presenting complaint:  flank pain, shortness of breath | | | | | | |
| Temp: 98.3 | HR: 108 | BP: 105/60 | | RR: 26 | O_2_Sat: 92% | FiO_2_:RA |
| Cap glucose: 92 | | | | GCS: (E V M ) 15 | | |
| Triage note:  55 y/o F with breast cancer presenting from hospice with sob, flank pain | | | | | | |
| Allergies: None | | | | | | |
| Past Medical History:   - Breast cancer s/p chemotherapy - Nephrolithiasis | | | Current Medications:   - Oxycodone-acetaminophen 2 tablets (5-325) PRN every 6 hours - Ibuprofen 400 mg every 8 hours - Docusate, senna | | | |

**Section 2B: Extra Patient Information**

| **A. Further History** | |
| --- | --- |
| *Include any relevant history not included in triage note above. What information will only be given to learners if they ask? Who will provide this information (mannequin’s voice, sim actors, SP, etc.)?*  55 year-old Female presenting from hospice with one day of worsening right-sided chest and flank pain associated with worsening shortness of breath. She experienced a mechanical trip and fall yesterday without LOC or head strike, witnessed by hospice staff with immediate return to baseline and no change in mental status. Patient has been taking her PRN percoset and ibuprofen with only minimal relief of her symptoms. Her husband noted worsening symptoms and when she was unable to speak in complete sentences they made the decision to come to the ED. The patient is able to shake her head yes or no to questions but can only provide one-word responses intermittently, and her husband provides additional history. She points to her right flank and chest when asked about pain and endorses shortness of breath. They deny fever/chills, other chest pain, abdominal pain, nausea or vomiting, change to bowel habits, urinary symptoms, or confusion. | |
| **B. Physical Exam** | |
| *List any pertinent positive and negative findings* | |
| Gen: female who appears older than stated age in moderate distress, grimacing, and uncomfortable | |
| Cardio: Tachycardic, regular, no M/R/G | Neuro: Non-focal, A+Ox3 |
| Resp: diminished breath sounds R mid to lower chest anteriorly and posteriorly, tachypneic | Head & Neck: supple, no tenderness |
| Abd: soft, non-tender, non-distended. No CVA tenderness | MSK/skin: ecchymosis to R flank |
|  | |

**Section 3: Technical Requirements/Room Vision**

| **A. Patient** |
| --- |
| ☒ Mannequin *(specify type and whether infant/child/adult): adult* |
| ☐ Standardized Patient |
| ☐ Task Trainer |
| ☐ Hybrid |
| **B. Special Equipment Required** |
| - Chest tube kit - Ultrasound |
| **C. Required Medications** |
| - Lidocaine - IV fentanyl, morphine, ketamine |
| **D. Moulage** |
| N/a |
| **E. Monitors at Case Onset** |
| ☐ Patient on monitor with vitals displayed  ☒ Patient not yet on monitor |
| **F. Patient Reactions and Exam** |
| *Include any relevant physical exam findings that require mannequin programming or cues from patient*  *(e.g. – abnormal breath sounds, moaning when RUQ palpated, etc.)*  A – patient speaking with one-word responses  B – diminished breath sounds R mid and lower fields  C – slightly tachycardic, no m/r/g  D – AEx4, GCS 15  E – ecchymosis and tenderness to R chest and flank, no spinal tenderness or step-offs, no other obvious injuries |

**Section 4: Sim Actor and Standardized Patients**

| **Sim Actor and Standardized Patient Roles and Scripts** | |
| --- | --- |
| *Role* | *Description of role, expected behavior, and key moments to intervene/prompt learners. Include any script required (including conveying patient information if patient is unable)* |
| Pt’s husband | The patient will be too uncomfortable and tachypneic, and she will convey that she wants decisions to be made by her husband. Husband (Mr Wilson) will be stressed and emotional, as pt recently placed in hospice and she is in significant pain after the fall.   - Must ask learners about options for pain medication - Prompt learners to provide basic explanation of hemothorax - Prompt learners to discuss chest tube placement if they do not prompt, risks and benefits - Prompt discussion of DNR/DNI status - pt is in hospice and DNR but husband can be hesitant regarding DNI if she worsens |

**Section 5: Scenario Progression**

| **Scenario States, Modifiers and Triggers** | | | | |
| --- | --- | --- | --- | --- |
| Patient State/Vitals | Patient Status | Learner Actions, Modifiers & Triggers to Move to Next State | | Facilitator Notes |
| **1. Baseline State**  Rhythm: sinus tach  HR: 105  BP: 105/60  RR: 26  O_2_SAT: 92 %  T: 36.8 ^o^C  GCS: 15 | Uncomfortable, moaning and intermittently groaning, alert and speaking only intermittently | Expected Learner Actions  ☐ IV/monitor  ☐ history from patient  ☐ history from husband  ☐ perform primary survey and physical exam  ☐ O2 via NC  ☐ may try to give pain medications  ☐ perform FAST or RUSH exam  ☐ portable CXR | Modifiers  *Changes to patient condition based on learner action*  - pt will vomit if PO medications given  Triggers  *For progression to next state*  - FAST (+) or cxr showing hemothorax  - | FAST/RUSH exam will show hemothorax R sided. Patient cannot tolerate anything PO and will vomit if given any tablet. |
| **2.**  Rhythm: sinus tach  HR: 115  BP: 93/55  RR: 22  O_2_SAT: 98 % (on NC)  T: 37^o^C  GCS: 14 | Will appear weak and close her eyes | Expected Learner Actions  ☐ 2^nd^ IV if not already established  ☐ start IVF bolus  ☐ transfuse at least 1 U uncrossed PRBC via rapid transfuser (ask pt’s husband about giving blood before)  ☐ ensure 2^nd^ type and screen is sent and order more blood | Modifiers  - if 2^nd^ type and screen not sent, blood bank will not release blood products  - BP will drop, HR elevate, pt will become more somnolent if no RBC given  Triggers  - blood pressure, HR will improve after blood transfusion | Husband should ask why patient begins looking worse, more unstable vitals |
| **3.**  Rhythm: sinus  HR: 95  BP: 110/60  RR: 20  O_2_SAT: 98 %  T: 37^o^C  GCS: 15 | Patient will still complain of pain | Expected Learner Actions  ☐ give pain meds (IV fentanyl, ketamine)  ☐ set up for chest tube (kit, pleur-evac, lido, scalpel, tube, sterile gown, drapes, gloves, etc., see below)  ☐ perform chest tube  ☐ call for CXR to confirm  ☐ discuss with trauma surgery, oncology for dispo  ☐ | Modifiers  - after discussion, husband and pt will be amenable to chest tube placement  - if lido not given pre chest tube, pt will yell in pain  Triggers  - Successful placement of chest tube  - Discussion of dispo with consults | Discuss chest tube placement with pt’s husband - discuss risks and benefits, pt’s wishes |

**Appendix A: Laboratory Results**

| CBC   WBC 15   Hgb 7.0 (11 from 5 days ago)   Plt 225  Lytes   Na 145   K 3.9   Cl 110   HCO_3_ 18   AG 12   Urea 20   Cr 0.8   Glucose 110  Extended Lytes   Ca 9   Mg 1.7   PO_4_ 3   Albumin 3.2   TSH wnl  VBG   pH 7.40   pCO_2_ 42   pO_2_ 40   HCO_3_ 20   Lactate 2.5 | Cardiac/Coags   Trop wnl   D-dimer elevated   INR wnl   aPTT wnl  Biliary   AST wnl   ALT wnl   GGT wnl   ALP wnl   Bili wnl   Lipase wnl  Tox   EtOH wnl   ASA wnl   Tylenol wnl   Dig level wnl   Osmols wnl  Other   B-HCG neg |
| --- | --- |

**Appendix B: ECGs, X-rays, Ultrasounds and Pictures**

| 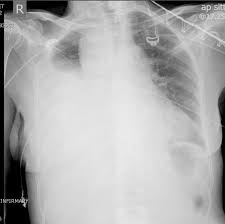  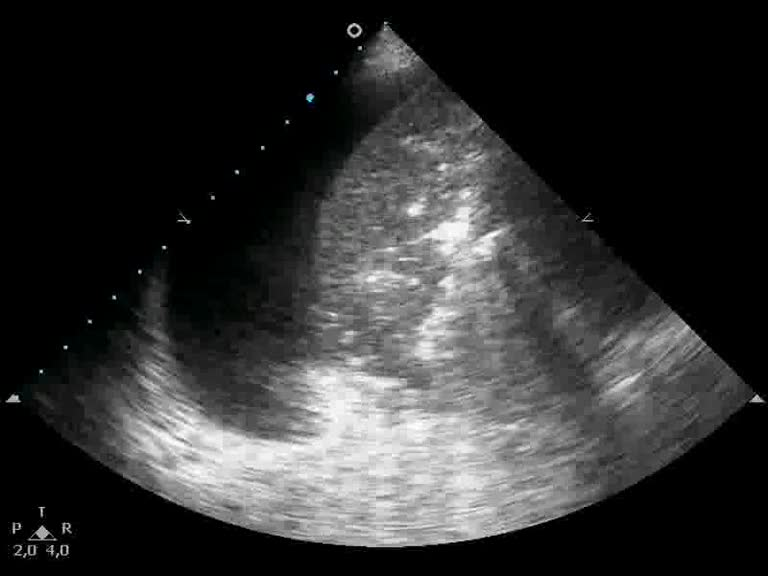  RUQ Ultrasound    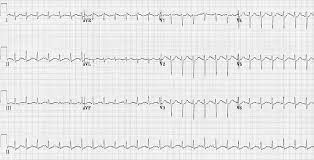 |
| --- |

SP Script

Hospice Case

| **Patient Name and DOB** | **Benjamin Wilson is the 58 year old husband of patient Melissa Wilson, age 55** |
| --- | --- |
| **Opening Statement/**  **Chief Complaint** | **“My chest hurts and I can’t breathe”** |
| **“Tell Me More About It”** | The patient is too short of breath and in too much discomfort to be able to answer. She defers history to you, her husband, as she is only able to shake her head yes or no to questions.  “My wife has been in hospice care for the past few weeks as her breast cancer has become advanced and not treatable, but has been stable for the past couple weeks. She tripped getting out of bed yesterday and fell to the ground but afterward seemed to be back to her baseline. She’s been taking her regular pain medications but today she couldn’t breathe and was in so much distress I couldn’t bear to see her continue to suffer and we had to come to the ER” |
| **Demeanor/ Physicality** | ***Worried, nervous, anxious*** |
| **History of Present Illness** | As mentioned, your wife has been in hospice for the past few weeks due to advanced breast cancer. She has been weak and tired but has been managing her pain well with her home Percoset and ibuprofen. Ms Wilson points to her right flank and chest when asked where the pain is. Besides the fall and worsening pain and shortness of breath today there have not been other falls, trauma, or other symptoms. |
| **Past Medical Hx** | ***Advanced metastatic breast cancer: diagnosed 3 months ago***  ***Nephrolithiasis (kidney stone): many years ago*** |
| **Past Surgical Hx** | None |
| **Allergies** | none |
| **Medications** | ***Percoset (oxycodone-acetaminophen) 5-325mg, 2 tablets every 6 hours as needed  (for pain)***  ***Ibuprofen 600mg, every 6 hours as needed (for pain)***  ***Docusate, senna daily (for constipation)*** |
| **Over the Counter/ Vitamins & Supplements** | ***None*** |
| **Social Hx** | ***Smoking: distant history, none in past 30 years***  ***Alcohol: none*** |
| **Family Medical Hx** | ***Parents: blood pressure (father), breast cancer (mother and grandmother), heart disease (father)***  ***Siblings: none***  ***Children: none*** |
| **ROS/Physical Findings** | ***No fevers, vomiting, altered mental status, diarrhea, other traumatic injury, or new weakness***  ***Female who appears older than stated age in distress, grimacing, uncomfortable, breathing quickly***  ***Bruising to R flank and chest*** |
| **Props/ Moulage** | ***SP should have hand-written med list*** |
| **SP Special instructions** | ***You are very involved in your wife’s care and you have been staying with her in the hospice facility for the past couple weeks.***  ***You both came to the difficult decision to place her in hospice recently but as her disease has now progressed rapidly and she has only been in hospice a couple weeks it has been difficult accepting that she will die soon.***  ***You will be very concerned about pain control for your wife, and if none is provided you will become more distressed and insist on urgent treatment. You will ask which medications are being given***  ***You have spoken extensively with your wife about her goals of care, and while she is DNR/DNI and under hospice care you are both amenable to treatment for situations like this, including a chest tube that would need to be placed emergently in this situation***  ***Your main concern is reducing suffering and pain for your wife. You discuss with the ER doctors about the potential risks and benefits of placing a chest tube emergently. It is a difficult decision to make as they are often quite uncomfortable and requires a stay in the hospital (specifically the Surgical Intensive Care Unit) , but it will help her breathing and ultimately you will want to proceed with it.***  ***You and the doctors discussed with Ms Wilson about the procedure, as she is in distress but still understands what is going on and can make a joint decision***  ***“Please help her”***  ***“What is going on? Why is she breathing so quickly?”***  ***“What is a hemathorax? What will happen if it’s not treated?”***  ***“Will the procedure cause her significant pain?”***  ***“What does this mean in terms of staying in the hospital and her hospice care?”*** |

**Section 1: Case Summary**

| **Scenario Title:** | **Pregnant Traumatic Arrest: Delivering Bad News** |
| --- | --- |
| Keywords: | Obstetric Emergencies, Traumatic Arrest, Hemopneumothorax |
| Brief Description of Case: | 23F G1P0 @ 32 weeks pregnant brought in front triage by boyfriend for “not acting right.”  Patient will be found to be altered with multiple signs of trauma all over body.  Patient will initially be tachycardic, tachypneic, and hypotensive due to hemorrhagic shock.  e-FAST will show a large left hemopneumothorax, and shortly afterwards patient will lose pulses. Team will start compressions (with lateral uterine displacement), and place a left  sided chest tube.  Patient will remain pulseless despite a chest tube and team will perform perimortem c-section. Patient will lose pulses again and will not be able to be resuscitated. Neonate will survive if resuscitated appropriately.   the ED team will break bad news to the boyfriend using SPIKES mnemonic. Further history taking will reflect patient fell down outside stairs while raining heavy |

| **Goals and Objectives** | |
| --- | --- |
| Educational Goal: | Recognize and manage traumatic injuries in the obstetrics patient |
| Objectives: | 1. Create a wide differential diagnosis for traumatic injuries in obstetric patients 2. Recognize signs and symptoms of hemothorax, and cardiac arrest 3. Initiate appropriate trauma resuscitation in an obstetric patient 4. Appropriately preform CPR in a pregnant patient, by laterally displacing the uterus during compressions 5. Appropriately place chest tube in a pregnant patient 6. Resuscitate a neonate 7. Deliver bad news to family members using spikes/remap method |

| **Learners, Setting and Personnel** | | | | | | |
| --- | --- | --- | --- | --- | --- | --- |
| Target Learners: | ☒ Junior Learners | | ☒ Senior Learners | | | ☐ Staff |
|  | ☐ Physicians | ☐ Nurses | | ☐ RTs | ☐ Inter-professional | |
|  | ☐ Other Learners: | | | | | |
| Location: | ☒ Sim Lab | | ☐ In Situ | | | ☐ Other: |
| Recommended Number of Facilitators: | Instructors: 2 | | | | | |
|  | Sim Actors:2 | | | | | |
|  | Sim Techs: 1 | | | | | |
|  |  |  |  |  |  |  |

**Section 2A: Initial Patient Information**

| **A.** **Patient Chart** | | | | | | |
| --- | --- | --- | --- | --- | --- | --- |
| Patient Name:  Jeanine Marcus | | | | Age: 23 | Gender: F | Weight: 70 kg |
| Presenting complaint: Confusion, SOB, Cough | | | | | | |
| Temp: 98.5 | HR:  126 | BP:  84/52 | | RR: 24 | O_2_Sat: 89% | FiO_2_:RA |
| Cap gluco se: 98 | | | | GCS: (E V M ) 2-3-4 = 9 | | |
| Triage note:    23F G1P0 @ 32 weeks pregnant brought in front triage by boyfriend for “not acting right.” Bruising noted to face, made surgical notification on arrival. | | | | | | |
| Allergies: NKDA | | | | | | |
| Past Medical History:  -   None | | | Current Medications:  -   Pre-Natal Vitamins | | | |
|  |  |  |  |  |  |  |

**Section 2B: Extra Patient Information**

| **A. Further History** | |
| --- | --- |
| *Include any relevant history not included in triage note above. What information will only be given to learners if they ask? Who will provide this information (mannequin’s voice, sim actors, SP, etc.)?*    The patient will be brought in by the boyfriend.  Ms. Marcus is an otherwise healthy 23 year old female, who is 38 weeks pregnant.  The boyfriend brought the patient in this afternoon because he noticed that the patient wasn’t acting right.  Upon being pressed further, he will relay that earlier this morning the patient fell down a flight of stairs.  After falling, she was unresponsive.  Boyfriend immediately decided to bring her to the hospital.  Boyfriend will be anxious, fearful when answering questions about events, though at the end of the case he will blame himself for the outcome as he was insisting they visit his elderly mother who lives in a dated building with a long set of stairs leading to the entrance. Patient/boyfriend live in a housing project in the Bronx wil large exterior stairs leading to the adjacent street. He will be  POBHx: G1P0, so far uncomplicated pregnancy, has had some prenatal care though has missed her last 2 visits. | |
| **B. Physical Exam** | |
| *List any pertinent positive and negative findings* | |
| Cardio: Tachycardic, regular rhythm, no M/R/G | Neuro: Confused though able to state name, anisocoria, withdraws to pain, unable to follow commands |
| Resp:  tachypneic, decreased breath sounds on the right, flail chest on the right | Head & Neck: Battle sign, peri-orbital ecchymosis, anisocoria, frontal contusion, abrasions over chin |
| Abdo: patch of ecchymosis over abdomen, firm, diffusely tender | MSK/skin: multiple ecchymotic lesions over trunk/extremities in various stages of healing |
| GU: No vaginal bleeding, no evidence of external trauma |  |
| Other: POCUS: L sided hemo-pneumothorax, + peritoneal free fluid, no pericardial effusion, +IUP with fetal movements, and fetal tachycardia | |

**Section 3: Technical Requirements/Room Vision**

| **A. Patient** |  | |
| --- | --- | --- |
| ☒ Mannequin *(specify type and whether infant/child/adult) Adult Pregnant Mannequin, Infant Mannequin* |  | |
| ☐ Standardized Patient |  | |
| ☐ Task Trainer |  | |
| ☐ Hybrid |  | |
| **B. Special Equipment Required** | |  |
| Chest tube/kit/chest wall model  Suction tubing and pleurevac  Intubation equipment | |  |
| **C. Required Medications** | |  |
| ACLS medications, RSI medications | |  |
| **D. Moulage** | |  |
| Bruising on mannequin | |  |
| **E. Monitors at Case Onset** | | |
| ☐ Patient on monitor with vitals displayed  ☒ Patient not yet on monitor | | |
| **F. Patient Reactions and Exam** | | |
| *Include any relevant physical exam findings that require mannequin programming or cues from patient*  A – intact, protecting airway  B – decreased breath sounds in the left, tachypneic, hypoxic, trachea deviated to right  C – tachycardic, hypotensive,  D – Anisocoria, GCS 2E-3V-4M (9),  E – Multiple signs of trauma: L sided flail chest, bruising to face/trunk/extremities, abrasions to chin, no active bleeding | | |
|  |  |  |

**Section 4: Sim Actor and Standardized Patients**

| **Sim Actor and Standardized Patient Roles and Scripts** | |
| --- | --- |
| *Role* | *Description of role, expected behavior, and key moments to intervene/prompt learners. Include any script required (including conveying patient information if patient is unable)* |
| Family member:  Boyfriend | Anxious, fearful, in distress. Earlier that day, boyfriend and patient were in an argument over his mother’s role in the child’s life. Mother was abusive to boyfriend, boyfriend wanted her involved regardless. Patient tired of the argument and decided to leave, she exited through the back of the building in a hurry. Weather was moderate rain, boyfriend was behind her yelling at her to come back. She slipped down the and fell down approx 12 steps, body stopped at a landing. Boyfriend will feel guilty, blame himself   - Will answer questions angrily - Will be able to answer questions accurately, closely involved with pregnancy, went to every OB appointment - Prototype type A, attempts to control everything only leading girlfriends death (motivation) |

**Section 5: Scenario Progression**

| **Scenario States, Modifiers and Triggers** | | | | |
| --- | --- | --- | --- | --- |
| Patient State/Vitals | Patient Status | Learner Actions, Modifiers & Triggers to Move to Next State | | Facilitator Notes |
| **1. Baseline State**  Rhythm: sinus tachycardia  HR: 126  BP: 84/52  RR: 30  O_2_SAT:89 %  T: 98.5^o^C  GCS: 9 | Thrashing around stretcher. Confused, tachypneic, hypoxic | Expected Learner Actions  ☐ Initiate Trauma resuscitation/Assess ABCs  ☐ Place on monitor  ☐ IV Access  ☐ Supplemental O2  ☐ e-FAST exam  ☐ Call for portable CXR  ☐ Transfuse Blood (MTP)  ☐ Analgesia  ☐ Perform needle thoracostomy  ☐ Set up and place chest right sided chest tube  ☐ Call trauma surgery  ☐ Call OB | Modifiers  *Changes to patient condition based on learner action*  - hypoxia will improve modestly with supplemental O2, chest tube placement  -Tachycardia will improve with transfusion, or chest tube placement  -hypotension will improve modestly with blood transfusion or chest tube  -  Triggers  *For progression to next state*   - Upon doing eFAST exam which shows the L sided hemothorax, and free fluid in abdomen the patient will become pulseless   - | eFAST will be positive for absent lung sliding on the left and fluid from hemothorax. eFAST will also be positive in RUQ.  OB and surgery will not come because they are busy on another case. |
| **2.**  Rhythm: PEA  HR: 0  BP: 0/0  RR: 0  O_2_SAT: 70%  T: 37^o^C  GCS: 3 | Patient goes into PEA arrest | Expected Learner Actions  ☐ Start ACLS  ☐ Intubate patient  ☐ Place chest tube  ☐ Perform perimortem c-section | Modifiers  - despite maximal therapy patient will not survive cardiac arrest. If the team starts perimortem within first few minutes of code neonate will survive  Triggers  -  - | If the fetus dies, the team will then have to deliver bad news to the baby’s father. |
| **3.** Rhythm: NSR  HR: 79  BP: 80/40  RR: 26  O_2_SAT: 70%  T: 37^o^C  GCS: 3  Neonate:  HR: 90  BP: 60/30  RR: 30  O2 SAT: 70% |  | ☐ Resuscitate neonate  ☐ Call peds and NICU  ☐ Deliver bad news to partner that mother and/or baby died  ☐ Call social work | Modifiers  - Neonate HR and sat will improve with PPV | Pregnant patient will not survive. Ideally, baby will survive.  Case will end when NICU and peds show up. |

**Appendix A: Laboratory Results**

| CBC   WBC 15   Hgb 7.3   Plt 150    Lytes   Na 146   K 3.8   Cl 110   HCO_3_ 20   AG 16   Urea 32   Cr 1.2   Glucose 145      ABG   pH 7.44   pCO_2_ 34   pO_2_ 45  Lactate 1.9    COVID: Negative  Flu A: Negative  Flu B: Negative | Cardiac/Coags   Trop wnl   D-dimer 23   INR wnl   aPTT wnl    Biliary   AST 74   ALT 96   ALP 150   Bili 1.0   Lipase 34  Type and Screen   A+ |
| --- | --- |

**Appendix B: ECGs, X-rays, Ultrasounds and Pictures**

| 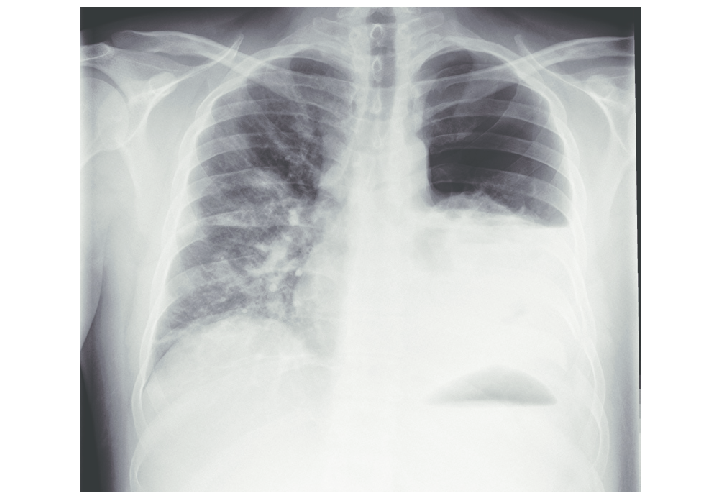           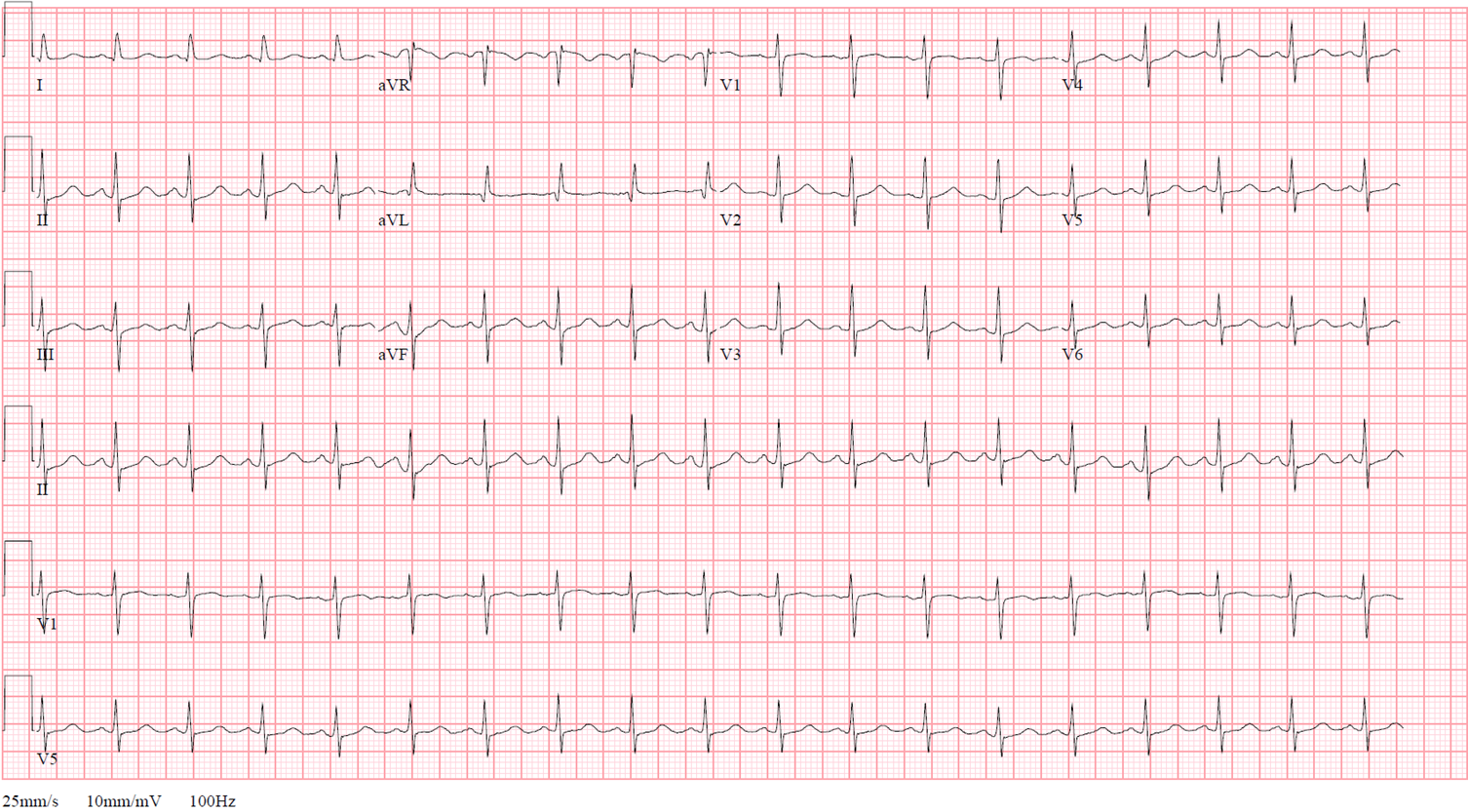 |
| --- |

**SP Case**

Delivering Bad News

| **Patient Name and DOB** | **Patient is Maria Carmine, 23 yo F, SP will play roll of partner, Anthony Mondell, 25 yo M** |
| --- | --- |
| **Opening Statement/**  **Chief Complaint** | **“Help my fiance!”** |
| **“Tell Me More About It”** | “We were arguing and she rushed down the stairs and fell” |
| **Demeanor/ Physicality** | ***Anxious, exasperated, fearful, crying*** |
| **History of Present Illness** | 23F hx distant appendectomy, currently G1 at 34 weeks. Had multiple episodes of vaginal bleeding early in pregnancy which resolved, uncomplicated course since then. Sonograms unremarkable up to this point |
| **Past Medical Hx** | Pt without relevant medical hx |
| **Past Surgical Hx** | Appendectomy at 10 years of age |
| **Allergies** | sulfa |
| **Medications** | None |
| **Over the Counter/ Vitamins & Supplements** | Prenatals |
| **Social Hx** | Pt and fiance had known each other for 4 years, and were very excited to become pregnant. Pt stopped using marijuana after becoming pregnant. Both families supportive in couple’s progress |
| **Family Medical Hx** | Breast Cancer in maternal aunt, father with heart disease |
| **ROS/Physical Findings** | ***N/A*** |
| **Props/ Moulage** | ***Ecchymosis over Left flank (FAST will be positive, rib fracture lacerating spleen causing hemorrhagic shock)*** |
| **SP Special instructions** | Earlier that day, boyfriend and patient were in an argument over his mother’s role in the child’s life. Mother was abusive to boyfriend, boyfriend wanted her involved regardless. Patient tired of the argument and decided to leave, she exited through the back of the building in a hurry. Weather was moderate rain, boyfriend was behind her yelling at her to come back. She slipped down and fell down approx 12 steps, body stopped at a landing. Fiance will feel guilty, blame himself   - Will answer questions angrily, “what does this have to do with my fiance??” “Don’t you already have all this in her chart??” - Will be able to answer questions accurately, closely involved with pregnancy, went to every OB appointment- “She’s exactly 34 weeks by ultrasound, she’s had the diabetes test which was normal, all of her blood pressures have been normal” - Prototype type A, “why is this happening to me?”   “What do you mean you had to cut her open??”  “Do you know what you’re doing?”  Medical team will attempt to save the mother, but will be unsuccessful. The baby will be saved. |
| **Door Chart** |  |
