# Supplementary figures and images for: Creation of a novel simulation based palliative care curriculum for the emergency medicine resident
Source: BMC Med Educ. 2026 May 25;26:1175. doi: 10.1186/s12909-026-09503-1 (PMC13383450; doi:10.1186/s12909-026-09503-1)

PEARLS Debriefing Framework (Eppich et al.)
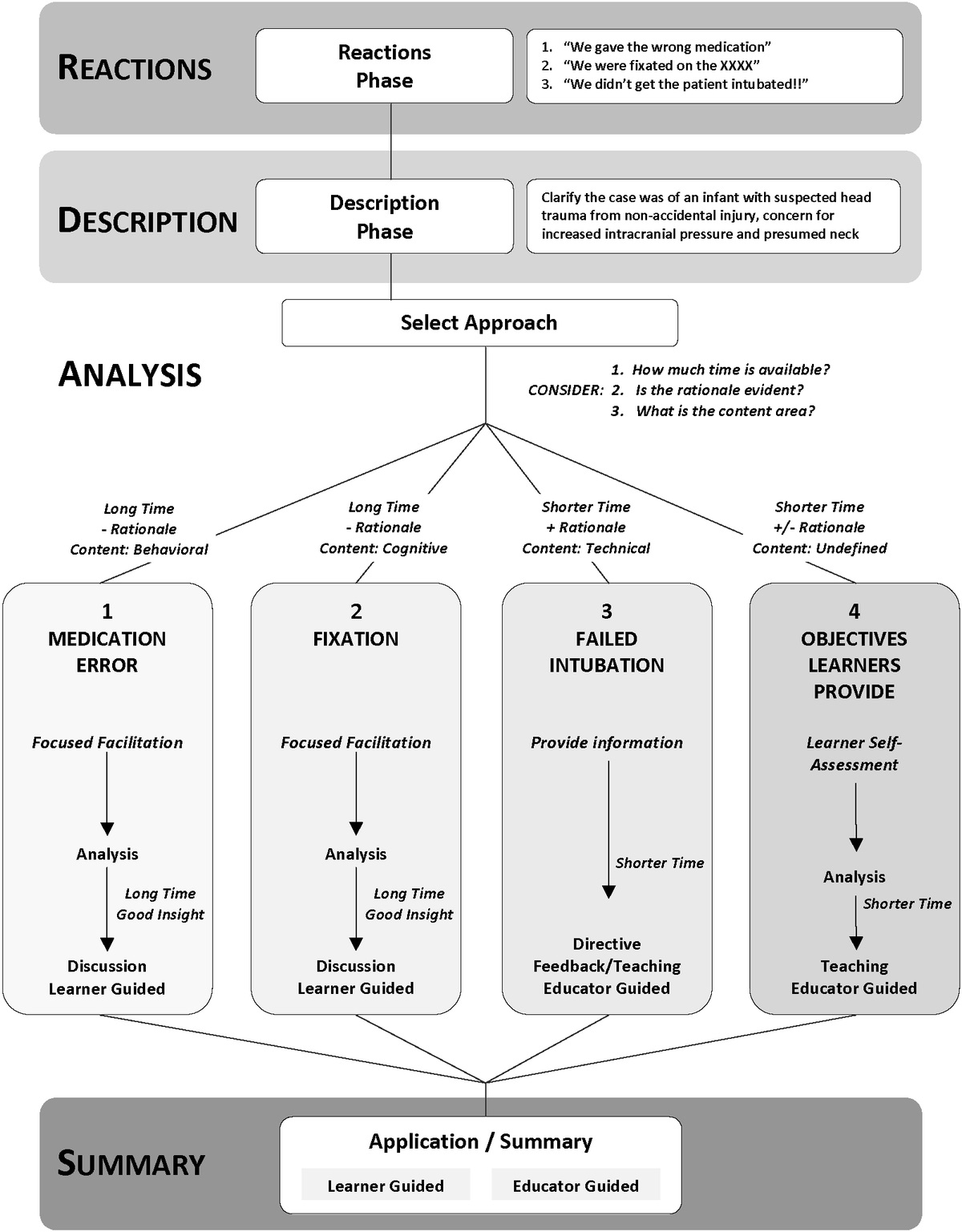

Supplement: Supplementary file 2 — Supplementary Material 2. [file 12909_2026_9503_MOESM2_ESM.docx]
