# Supplementary material for: Creation of a novel simulation based palliative care curriculum for the emergency medicine resident
Source: BMC Med Educ. 2026 May 25;26:1175. doi: 10.1186/s12909-026-09503-1 (PMC13383450; doi:10.1186/s12909-026-09503-1)
Supplement: Supplementary file 3 — Supplementary Material 3. [file 12909_2026_9503_MOESM3_ESM.docx]

Palliative Care for the Emergency Medicine Resident

**Section 1: Demographics**

1. Gender (Mark only one oval)
   1. Male
   2. Transgender Male
   3. Female
   4. Transgender Female
   5. Non-Binary
   6. Prefer Not to Say
   7. Other
2. Unique Identifier: Please enter the last 2 digits of your birth year and the first 3

letters of the street you grew up on.

I.e. if you were born in 1995 and grew up on Smith Street your identifier would be

95Smi
_______________________________________________________________________

1. How old are you?

_______________________________________________________________________

1. What Degree Do You Hold? (select all that apply)
   1. MD
   2. DO
   3. MBA
   4. MPH
   5. Other
2. What Religion Do You Identify As? (please select one)
   1. Judaism
   2. Christianity
   3. Catholicism
   4. Muslim
   5. Other
3. Level of Training (please select one)
   1. PGY1
   2. PGY2
   3. PGY3
   4. PGY4
4. In the last 3 months approximately how many palliative care conversations (think

goals of care, advanced directives, code status) have you personally led? (please select one)

- 1. None
  2. 1-5
  3. 6-10
  4. 11-15
  5. >15

1. Have you ever received any formal training in palliative care? (Lectures, workshops,

etc.)

- 1. Yes
  2. No

1. If you said yes to the previous question, please describe the experience of palliative

care training below (e.g. what did the training entail, how long was it)
_______________________________________________________________________

**Section 2: Attitudes Towards and Practice Patterns of Palliative Care**

Please pick the option that best fits your opinion on the following statements. All statements are graded on a linear scale where:

1= strongly disagree

2=disagree

3=neutral

4=agree

5=strongly agree

1. I feel comfortable notifying family members and loved ones of a deceased patient
   1. Strongly Disagree
   2. Disagree
   3. Neutral
   4. Agree
   5. Strongly Agree
2. I routinely discuss advanced directives with my patients.
   1. Strongly Disagree
   2. Disagree
   3. Neutral
   4. Agree
   5. Strongly Agree
3. I feel comfortable having code status and goals of care discussions.
   1. Strongly Disagree
   2. Disagree
   3. Neutral
   4. Agree
   5. Strongly Agree
4. I frequently consult palliative care for assistance with advanced illness

complications and end of life care

- 1. Strongly Disagree
  2. Disagree
  3. Neutral
  4. Agree
  5. Strongly Agree

1. Emergency Physicians Play an Important Role in Palliative Care
   1. Strongly Disagree
   2. Disagree
   3. Neutral
   4. Agree
   5. Strongly Agree
2. Palliative care is indicated for any patient with a life-threatening diagnosis only if

they have complex symptoms such as pain, dyspnea…etc.

- 1. Strongly Disagree
  2. Disagree
  3. Neutral
  4. Agree
  5. Strongly Agree

1. Patients or families are often unwilling or unready to elect palliative care

services.

- 1. Strongly Disagree
  2. Disagree
  3. Neutral
  4. Agree
  5. Strongly Agree

1. All adults and children who are terminally ill are candidates for palliative care

services, not just those with cancer

- 1. Strongly Disagree
  2. Disagree
  3. Neutral
  4. Agree
  5. Strongly Agree

1. Palliative care aims to enhance quality of life for the patient and family.
   1. Strongly Disagree
   2. Disagree
   3. Neutral
   4. Agree
   5. Strongly Agree
2. Palliative care includes skilled care for terminally ill patients.
   1. Strongly Disagree
   2. Disagree
   3. Neutral
   4. Agree
   5. Strongly Agree
3. Palliative care includes expert pain and symptom management.
   1. Strongly Disagree
   2. Disagree
   3. Neutral
   4. Agree
   5. Strongly Agree

**Section 3: Knowledge About Palliative Care**

What follows are 3 general questions and 13 MCQs regarding palliative care.

If you do not know the answer, please select I don’t know rather than guessing.

1. How would you rate your experience in managing pain in cancer and palliative

patients?

- 1. Non-Existent
  2. Weak
  3. Good
  4. Very Good
  5. Excellent

1. How would you rate your experience in managing other palliative care symptoms

(constipation, nausea and vomiting, anorexia, etc.)?

- 1. Non-Existent
  2. Weak
  3. Good
  4. Very Good
  5. Excellent

1. How would you rate your experience in conducting family counseling and breaking

bad news?

- 1. Non-Existent
  2. Weak
  3. Good
  4. Very Good
  5. Excellent

1. Palliative care is different from traditional care because palliative care: Is curative
   1. Is equivalent to hospice care
   2. Is focused on comfort rather than cure
   3. Is equivalent to end of life care
   4. Withdraws care
   5. I don’t know
2. Which of the following members of the healthcare team are important to the

delivery of palliative care?

- 1. Physicians
  2. Nurses
  3. Dieticians
  4. Physical Therapists
  5. Occupational Therapists
  6. All of the Above
  7. I don’t know

1. A 65-year-old male with metastatic prostate Ca presents with severe back pain.

He ran out of his home meds (oxycodone 5mg Q8hr) and is requesting a refill. The pharmacy is out of oxycodone and is requesting an alternative. Which is equivalent?

- 1. Dilaudid 2.5 mg PO
  2. Fentanyl Patch 25 mcg
  3. Morphine 7.5 mg PO
  4. Morphine 15 mg PO
  5. I don’t know

1. Which is the most appropriate drug can be used in management of delirium in

palliative care:

- 1. Fentanyl
  2. Haloperidol
  3. Midazolam
  4. Ondansetron
  5. Paroxetine
  6. I don’t know

1. Dyspnea related to advanced lung cancer can be best treated by:
   1. Morphine
   2. Midazolam
   3. Dexamethasone
   4. Oxygen mask and Albuterol
   5. 1 + 2
   6. I don’t know
2. Regarding Hypercalcemia in cancer patients, it:
   1. Is the most common life threating metastatic disorder in cancer patients
   2. Needs to be treated if adjusted serum calcium ≥3.5 mmol/l or the patient is

symptomatic.

- 1. Is Related to bone cancer only.
  2. Can cause severe diarrhea.
  3. All of the above
  4. I don’t know

1. The hallmarks of opioid toxicity are all the following except:
   1. Oxygen saturation ≤ 90%
   2. Respiratory rate ≤ 10 /min
   3. Pinpoint pupils
   4. Sedation
   5. Jerky movements
   6. I don’t know
2. Signs of superior vena caval obstruction are all the following except:
   1. Cyanosis
   2. Pulsatile distended neck veins
   3. Edema in the hands
   4. Dilated veins over the chest wall
   5. Periorbital edema
   6. I don't know
3. Management of Catastrophic bleeding in palliative care includes
   1. Midazolam
   2. Vitamin K
   3. Dark towel
   4. Tranexamic injection in large doses.
   5. 1+3
   6. I don't know
4. Treatment of metastatic spinal cord compression include the following
   1. High dose dexamethasone
   2. Neurosurgical intervention
   3. Chemotherapy
   4. Radiation therapy
   5. All of the above
   6. I don’t know
5. All of the following are characteristics of oral opioid analgesics except:
   1. They are effective for localized and generalized pain
   2. They are easily administered
   3. There is stigma and fears associated with use
   4. There is a ceiling effect to analgesia
   5. None of the above
   6. I don't Know
6. In the State of New York, which of the following lists the correct order of

priority for surrogate decision makers in the event that a patient does not have the

capacity to make their own medical decisions?

- 1. Adult child > sibling > spouse > parent > close friend > court appointed guardian
  2. Spouse > parent > adult child > sibling > court-appointed guardian > close friend
  3. Court-appointed guardian > spouse > adult child > parent > sibling > close friend
  4. Adult child > spouse > court-appointed guardian > parent > sibling > close friend
  5. Parent > spouse > court-appointed guardian > close friend > sibling
  6. I don’t know

1. In order to demonstrate decision-making capacity, the patient should have

which of the following:

- 1. Ability to evaluate risks and benefits and appreciate their consequences
  2. Ability to receive and understand information about medical care
  3. Ability to communicate one’s decision to the physician
  4. All of the above
  5. I don’t know
